# EDA-Fibronectin Originating from Osteoblasts Inhibits the Immune Response against Cancer

**DOI:** 10.1371/journal.pbio.1002562

**Published:** 2016-09-21

**Authors:** Stephanie Rossnagl, Eva Altrock, Carla Sens, Sabrina Kraft, Katrin Rau, Michael D. Milsom, Thomas Giese, Yvonne Samstag, Inaam A. Nakchbandi

**Affiliations:** 1 Max-Planck Institute of Biochemistry, Martinsried, Germany; 2 Institute of Immunology, University of Heidelberg, Heidelberg, Germany; 3 German Cancer Research Center (DKFZ), Division of Stem Cells and Cancer, Experimental Hematology Group, and Heidelberg Institute for Stem Cell Technology and Experimental Medicine, gGmbH (HI-STEM), Heidelberg, Germany; Royal Veterinary College London, UNITED KINGDOM

## Abstract

Osteoblasts lining the inner surface of bone support hematopoietic stem cell differentiation by virtue of proximity to the bone marrow. The osteoblasts also modify their own differentiation by producing various isoforms of fibronectin (FN). Despite evidence for immune regulation by osteoblasts, there is limited knowledge of how osteoblasts modulate cells of the immune system. Here, we show that extra domain A (EDA)-FN produced by osteoblasts increases arginase production in myeloid-derived cells, and we identify α5β1 as the mediating receptor. In different mouse models of cancer, osteoblasts or EDA-FN was found to up-regulate arginase-1 expression in myeloid-derived cells, resulting in increased cancer growth. This harmful effect can be reduced by interfering with the integrin α5β1 receptor or inhibiting arginase. Conversely, in tissue injury, the expression of arginase-1 is normally beneficial as it dampens the immune response to allow wound healing. We show that EDA-FN protects against excessive fibrotic tissue formation in a liver fibrosis model. Our results establish an immune regulatory function for EDA-FN originating from the osteoblasts and identify new avenues for enhancing the immune reaction against cancer.

## Abstract 2

Osteoblasts produce an isoform of fibronectin (EDA-fibronectin) that acts on myeloid cells to increase arginase-1 expression and protect against fibrosis. However, it can also enhance cancer growth; interfering with the interaction between EDA-fibronectin and its receptor diminishes this effect.

## Author Summary

Osteoblasts, which are the cells that produce bone, line the inner surface of the bone and are adjacent to the marrow that generates all the different blood cells. Osteoblasts have a close relationship with hematopoiesis, and it has been shown that a transient elimination of osteoblasts leads to the decrease of hematopoietic stem cells and progenitor cells. Fibronectin (FN) is an extracellular matrix protein with a known role in hematopoiesis in vitro that is secreted by osteoblasts. Here, we analyze the role of FN in hematopoiesis and find that an isoform that contains the extra domain A (EDA) and is produced by the osteoblasts affects both the number and future behavior of a subset of immune cells. EDA-FN protects against excessive fibrotic tissue formation in a liver fibrosis model. The same process, however, is detrimental in cancer, because it prevents the organism from mounting a potent immune response against the cancer and induces an increase of cancer growth. Mechanistically, we find that the EDA domain binds to the cell surface receptor α5β1 integrin and enhances the production of the anti-inflammatory and immunosuppressive factor arginase-1. We conclude that EDA-FN production by osteoblasts modulates immune cell behavior, and that interfering with this mechanism opens up new possibilities for enhancing an immune reaction against cancer.

## Introduction

The inner surface of the bone is lined with preosteoblasts and osteoblasts in the immediate vicinity of bone marrow. Hematopoietic stem cells are found close to the bone lining cells, which represent the osteoblastic or endosteal niche as well as the vascular niche [1,2]. Several groups have reported a relationship between osteoblasts and hematopoiesis [3–5]. These findings culminated in experimental evidence showing that temporary destruction of the osteoblasts led to loss of hematopoietic stem cells as well as various hematopoietic progenitor cells [6]. Osteoblasts produce a variety of cytokines that affect hematopoiesis, such as interleukin-6, and respond to these same cytokines [7–11]. In line with this, stimulating the osteoblasts with a bone-active hormone called parathyroid hormone led to an increase in myeloid cells in the bone marrow [11].

Osteoblasts also secrete fibronectin (FN), a ubiquitously expressed extracellular matrix protein produced by various cell types in mammals. FN supports several vital functions such as differentiation [12–16], migration [12,17], homing of bone marrow stem cells [18], and hematopoiesis in vitro [19]. Its ability to affect opposite functions, such as maintaining stemness [20] or enhancing differentiation of progenitor cells, depends on the receptors involved [12] and is mediated by the presence of several isoforms containing or lacking extra-domains-A (EDA) and/or B (EDB) and by other forms of alternative splicing, as well as posttranslational modifications [21]. The presence of the EDA, for example, allows binding to α4β1 and α9β1 integrin [21] and enhances binding of FN to α5β1 [22]. Although most FN isoforms also contain the CS1 domain, which binds to α4β1, and all isoforms contain the arginine-glycine-aspartic acid (RGD) sequence, which binds to α5β1 integrin, the characteristics of binding to integrins as well as signaling and biological consequences are clearly changed by the presence of the EDA-domain [22,23]. Both α4- and α5-containing integrins are expressed in the bone marrow. α4-containing integrin is expressed on the earliest progenitors in the bone marrow and has been implicated in homing of immune cells to the gut and bone marrow and, hence, inflammation [24–28]. Its role in myelopoiesis seems limited and controversial, however [20,29,30]. Both α4β1 and α5β1 affect the proliferation, apoptosis, and differentiation of erythroid and lymphocytic cells [19,29–31]. Thus, EDA-containing FN could be responsible for some of the effects attributed to FN in hematopoiesis [20]. Indeed, EDA-containing FN is produced by osteoblasts together with EDB-containing FN. These isoforms are required to enhance osteoblast differentiation, and this effect might be at least partially mediated by α4β1 integrin [14]. Although these associations suggest that EDA-containing FN originating from the osteoblasts might play a role in hematopoiesis, this question has not been examined in the past.

The aim of this study was to determine whether FN originating from the osteoblasts affected hematopoiesis, which isoform and receptor are involved, and which consequences ensue for the organism.

## Results

### Depletion of FN in Osteoblasts Affects Myelopoiesis

**Fig 1:**
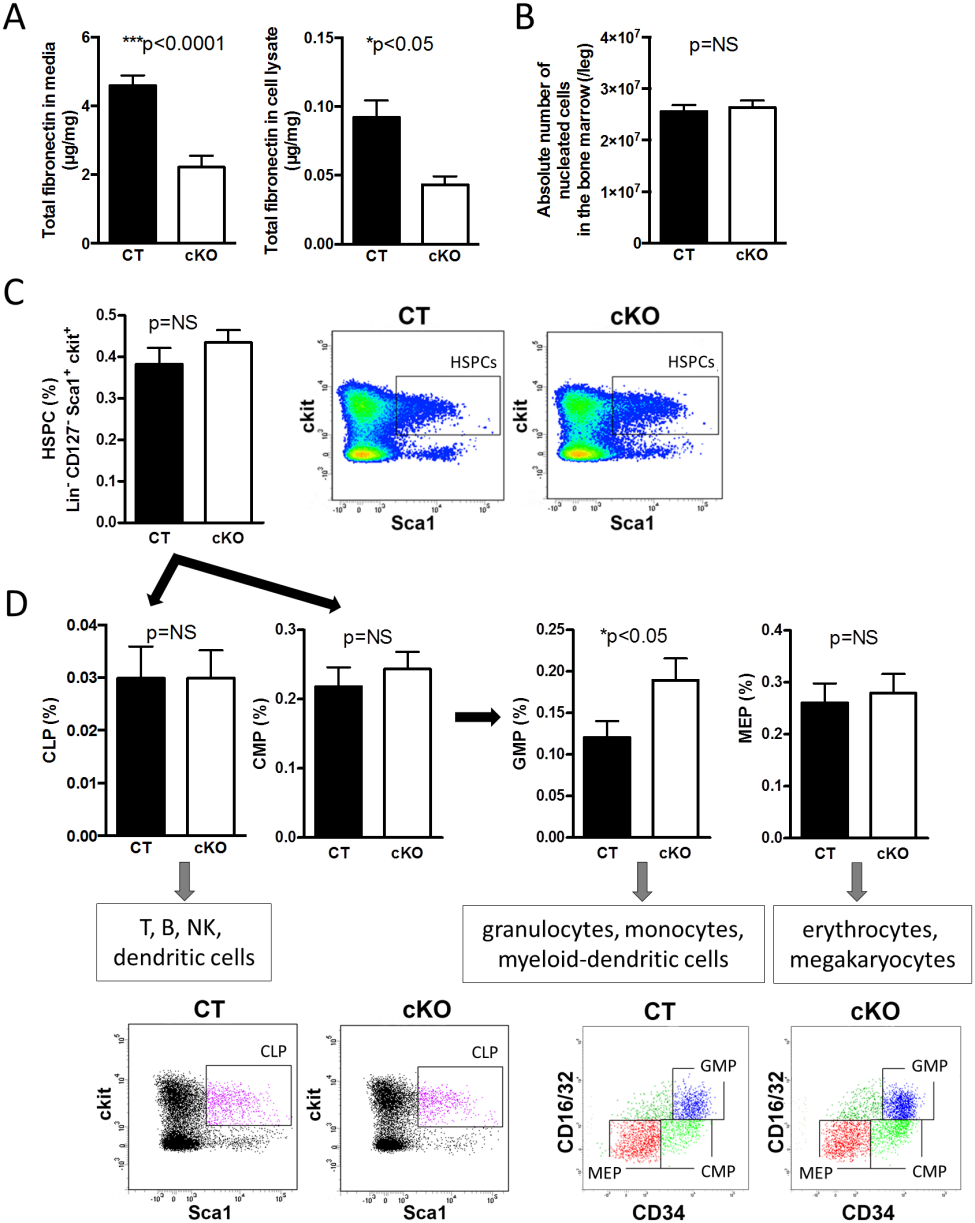
Depletion of FN in osteoblasts affects myelopoiesis. (A) Total FN is diminished in conditioned media and cell lysates of isolated primary osteoblasts. CT: control, cKO: conditional knockout mice with depletion of FN production in the osteoblasts, *n* = 19/18 and 23/16 in seven experiments. (B) There was no difference in bone marrow cellularity, *n* = 17/18 in seven experiments. (C,D) Only GMPs are increased in cKO bone marrow, but no changes are found in HSPCs (Hematopoietic-stem-and-progenitor-cells), common myeloid progenitors (CMP), common lymphoid progenitors (CLP), or megakaryocyte–erythroid progenitors (MEP), *n* = 20/18 in six experiments except HSPCs *n* = 28/28 in eight experiments. Student’s *t* tests were performed for comparisons. Underlying data for A–D are provided in S1 Data.

**Fig 2:**
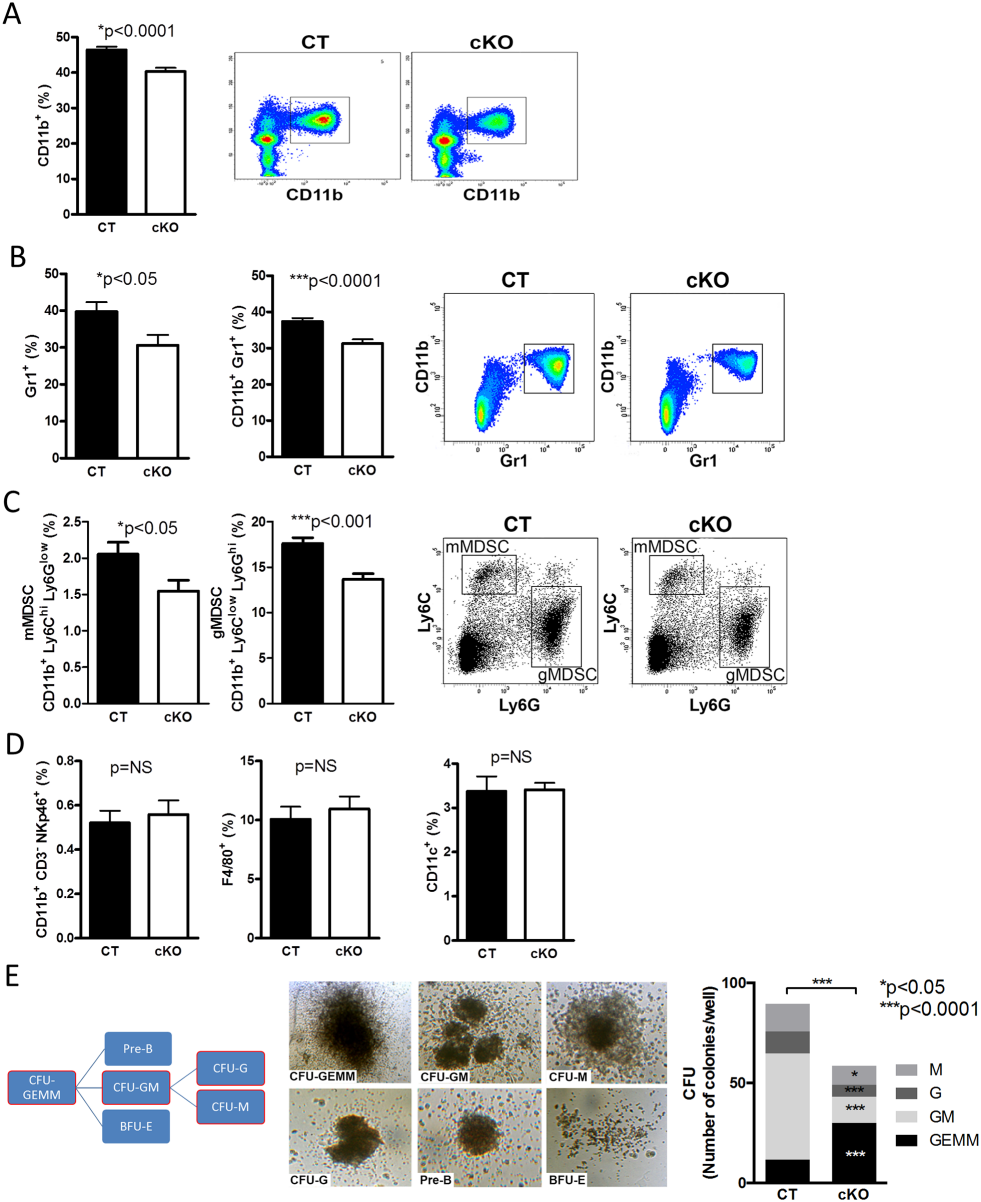
Changes in myeloid populations. (A) Myeloid cells (CD11b^+^) are diminished in the bone marrow in the absence of FN in osteoblasts. (B,C) Populations originating from myeloid cells were reduced in the bone marrow (Gr1^+^, CD11b^+^Gr1^+^, myeloid-derived suppressor cells; mMDSC, gMDSC), *n* = 15/17 biological replicates in five experiments. (D) No changes in natural killer cells (CD11b^+^ CD3^-^ NKp46^+^), macrophages (F4/80^+^), or dendritic cells (CD11c^+^) were detected, *n* = 10/10 biological replicates in three experiments. Student’s *t* tests were used for comparisons. (E) Counting the colonies (CFUs: Colony-forming units) formed in vitro showed increased numbers of the colonies reflecting the progenitors (GEMM) and decreased colonies along the myeloid lineage (GM: granulocyte/monocyte, G: granulocyte, M: monocyte), *n* = 20/20 biological replicates in five experiments. Student’s *t* tests were performed for all individual CFU populations and the total number. Underlying data for A–E are provided in S1 Data.

**Fig 3:**
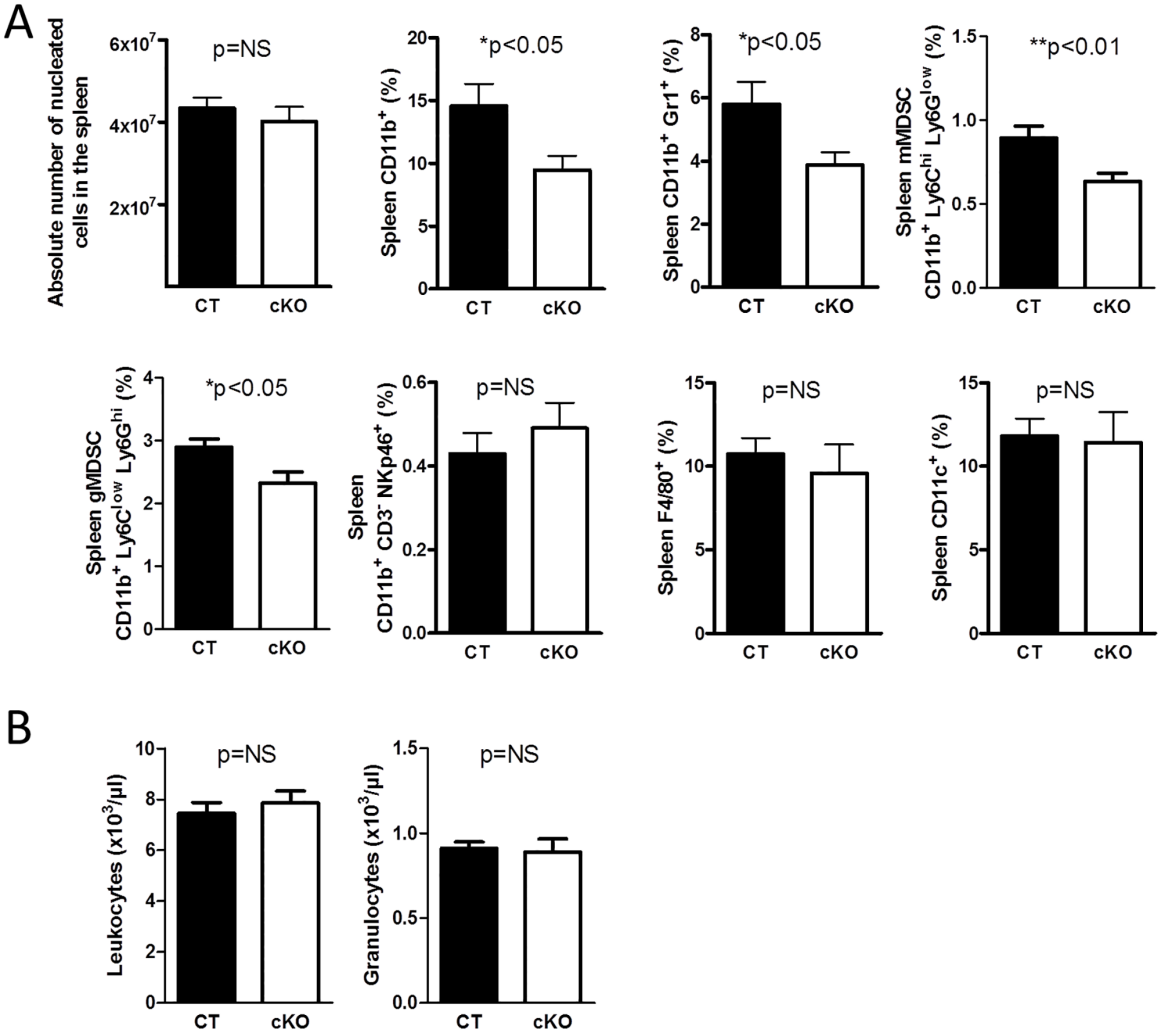
Changes in spleen and peripheral blood. (A) There was no difference in spleen cellularity, but the same myeloid populations affected in the bone marrow were diminished in the spleen, *n* = 10/11 biological replicates in three experiments. (B) Evaluation of peripheral blood cells revealed no difference between control (CT) and conditional knockout mice (cKO). Shown are only leukocytes (WBC) and granulocytes count, *n* = 5/6 in two experiments. Student’s *t* tests were performed for comparisons. Underlying data for A and B are provided in S1 Data.

FN supports hematopoiesis in vitro [19,20]. In order to determine whether FN originating from osteoblasts affects hematopoiesis, conditional knockout mice (cKO) were generated in which FN was depleted in differentiating osteoblasts (using the cre/loxP system by means of the collagen-α1(I)-promoter attached to cre in mice homozygote for the floxed FN gene (Col-cre_FN^fl/fl^) and compared to littermate controls (CT: FN^fl/fl^) (Fig 1A) [14]. This results in a delay in osteoblast differentiation as reported by us [14] but does not affect bone mineral density, osteoclasts, adipocyte numbers, or sinusoid area (S1 Fig). Despite normal cellularity in the bone marrow (Fig 1B), we evaluated the relationship of the various hematopoietic populations (Figs 1C, 1D, 2A–2D and S2A Fig). We detected increased percentages of granulocyte-monocyte-progenitors (GMPs) in the bone marrow of cKO by flow cytometry (Fig 1D). Further characterization showed decreased myeloid cells (CD11b^+^) as well as CD11b^+^Gr1^+^-cells in cKO (Fig 2A and 2B). The two subpopulations of myeloid-derived suppressor cells (MDSCs) (monocytic MDSC [mMDSC]: CD11b^+^ly6C^high^Ly6G^low^ and granulocytic MDSC [gMDSC]: CD11b^+^ly6C^low^Ly6G^high^) were diminished in cKO (Fig 2C) even when α4 integrin expression (CD49d) (CD49d^+^ in mMDSC and CD49d^-^ in gMDSC) was included (S2B Fig) [32]. No difference was detected in natural killer cells, macrophages, dendritic cells, various blood cell populations (Fig 2D and S2C Fig), or the osteoclasts, which develop from myeloid cells as determined by histomorphometry (S1B Fig). Differentiation of bone marrow colony-forming units (CFUs) in culture revealed an overall lower number of colonies in cKO with increased numbers of progenitor cells (CFU-granulocyte/erythrocyte/monocyte/megakaryocyte [CFU-GEMM]) and reduced myeloid populations (CFU-granulocyte/monocyte [CFU-GM], CFU-granulocyte [CFU-G], CFU-monocyte [CFU-M]) (Fig 2E). Similar changes were seen in the spleen despite normal absolute cell numbers (Fig 3A and S2C Fig). Peripheral blood cell counts were not affected, however (Fig 3B).

In summary, depletion of FN in osteoblasts is associated with diminished differentiation of precursor cells to myeloid cells.

### FN Produced by the Osteoblasts Increases CD11b^+^-Cells in the Bone Marrow

We next examined whether diminished myeloid cells were directly attributable to loss of osteoblast FN both in vivo and in vitro.

**Fig 4:**
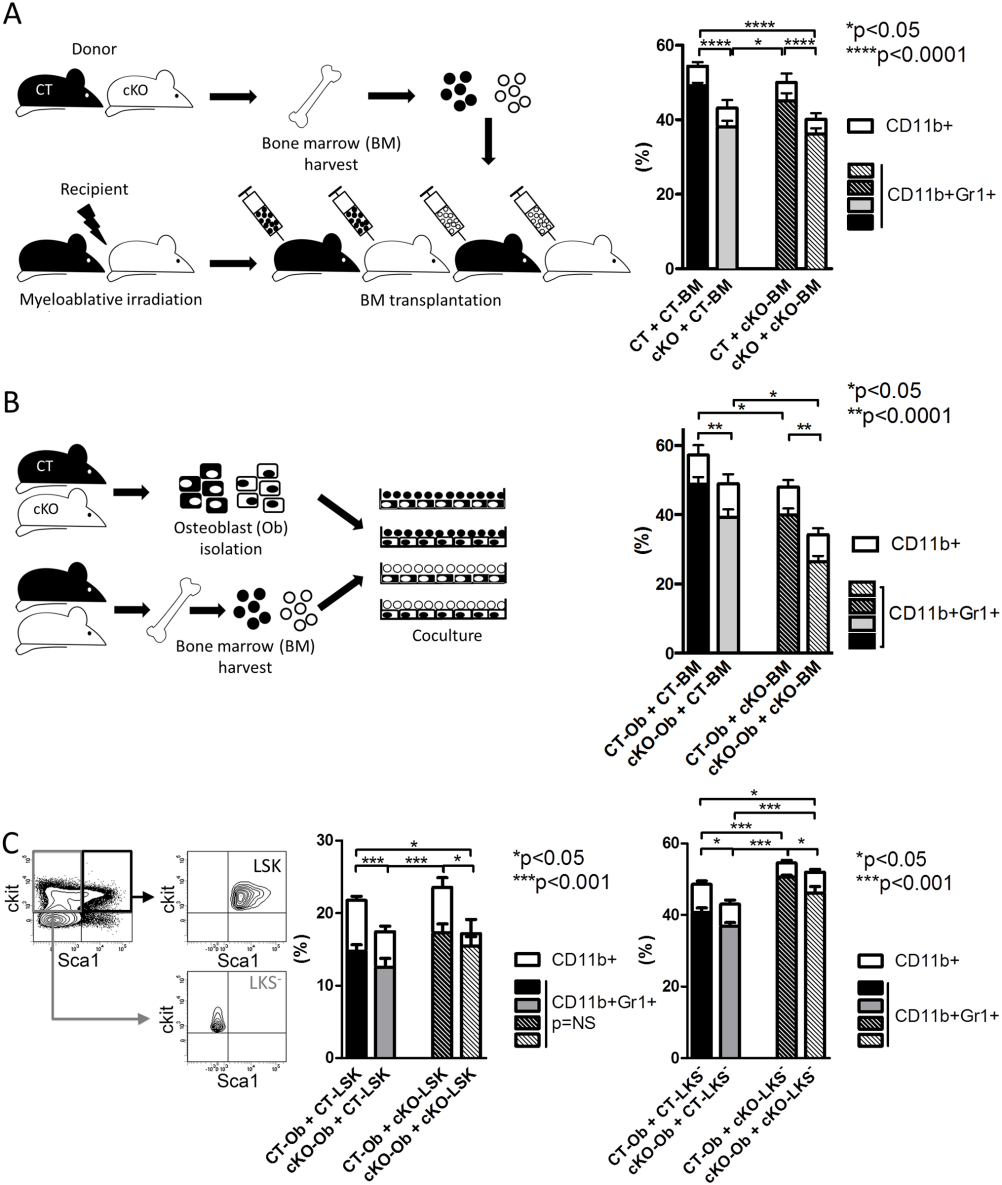
FN produced by the osteoblasts increases CD11b^+^-cells in the bone marrow. (A) Transplantation of CT and cKO bone marrow in CT and cKO animals generated four groups and showed recovery of decreased myeloid cells in cKO bone marrow when transplanted in CT, whereas CT bone marrow showed a decrease in myeloid cells when transplanted into cKO animals, *n* = 10/9/11/13 biological replicates in four experiments. (B) Coculture of isolated osteoblasts with bone marrow showed a significant decrease in myeloid cells when osteoblasts are unable to produce FN, *n* = 23/37/20/29 replicates in five experiments. (C) Coculture of isolated osteoblasts with sorted LSK (lineage^-^c-kit^+^sca-1^+^, which are hematopoietic stem and progenitor cells: HSPCs) or LKS^-^ (lineage^-^c-kit^+^sca-1^-^, which include GMPs, CMPs, and MEPs) also showed decreased differentiation of CD11b^+^ myeloid cells in the presence of osteoblasts unable to produce FN. LSK and LKS^-^ populations before and after sorting are shown on the left, *n* = 14/11/9/11 and 9/10/9/9 biological replicates in three experiments. ANOVA followed by *t* tests for the comparisons between the individual groups was performed. Underlying data for A–C are provided in S1 Data.

First, transplantation experiments were performed and myeloid cells evaluated in vivo. Mice were lethally irradiated and injected with 10^6^ bone marrow cells. CT and cKO bone marrow was transplanted into CT and cKO mice, generating four experimental groups. Both CT and cKO bone marrow transplanted to cKO mice showed a decrease in myeloid cells after 4 wk, whereas cKO bone marrow recovered almost completely when transplanted into CT mice with regard to CD11b^+^- or CD11b^+^Gr1^+^-cells (Fig 4A). There were no differences in the recovery of peripheral leukocytes (S3 Fig).

We next evaluated in vitro the effect of isolated osteoblasts on bone marrow differentiation. Isolated osteoblasts from CT and cKO animals were cultured, and CT or cKO bone marrow was added. After 24 h of coculture, cKO osteoblasts were associated with fewer myeloid cells than CT osteoblasts, irrespective of whether CT or cKO bone marrow was used (Fig 4B). Because, however, the differentiation of myeloid cells was lower when cKO bone marrow was used (Fig 4B, right two columns), presumably because of already diminished CD11b^+^-cells in the isolated cKO bone marrow, we proceeded with evaluation of the effect of coculture of osteoblasts with defined stem and progenitor populations. For this, we sorted two populations of hematopoietic cells: a population called hematopoietic stem and progenitor cells (HSPCs) as defined by Lineage^-^c-kit^+^*sca-1*^+^ (LSK) and a population containing the progenitor cells GMP, CMP, and MEP as defined by Lineage^-^c-kit^+^*sca-1*^-^ (LKS^-^) (Fig 4C and S2A Fig) [33,34]. Both populations (LSK and LKS^-^) resulted in fewer myeloid cells when cocultured with cKO osteoblasts compared to coculture with CT osteoblasts.

Consequently, the decrease in myeloid cells in cKO is caused by FN depletion in osteoblasts.

### EDA-Containing FN Augments Myeloid Cells In Vitro

**Fig 5:**
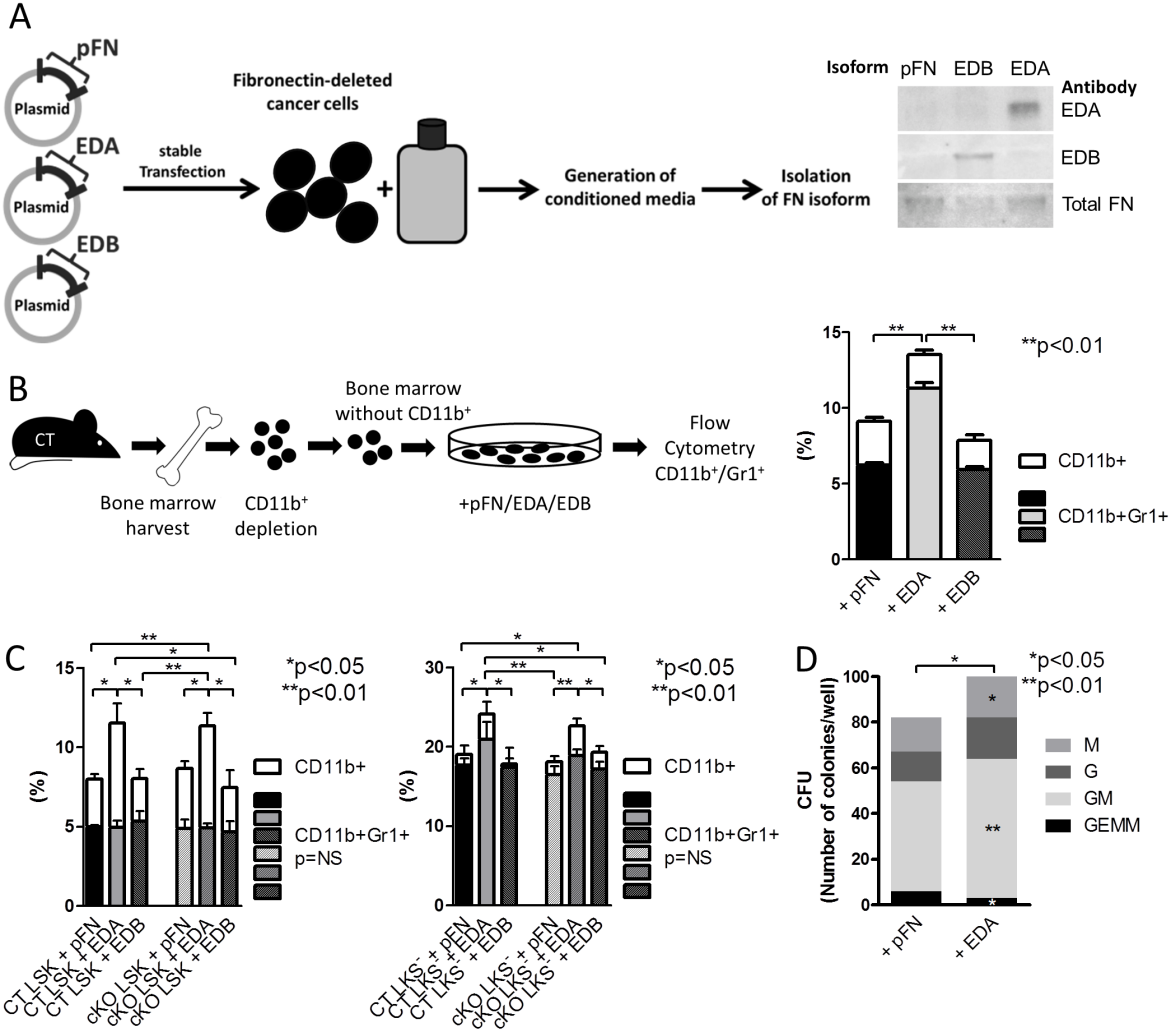
EDA-containing FN augments myeloid cells in vitro. (A) Constructs to produce EDA, EDB, and pFN isoforms were generated and stably transfected into a cancer cell line in which FN had been deleted using 5ʹ UTR-specific shRNA. Media from these cells were collected and the isoforms isolated. Western blotting confirmed the presence of EDA in FN isolated from EDA- but not pFN- or EDB-transfected cells. (B) Depleting bone marrow of CD11b^+^-cells and culturing the depleted bone marrow with pFN, EDA, or EDB showed that only EDA increased CD11b^+^, *n* = 13/13/10 biological replicates in four experiments. ANOVA followed by *t* tests was used for statistical analysis. (C) Sorted HSPCs as defined by LSK and a population enriched with the progenitor populations GMP, CMP and MEP as defined by LKS^-^ were cultured with FN isoforms pFN, EDA, and EDB. Again, only EDA showed a stimulating effect on the differentiation of CD11b^+^ myeloid cells, *n* = 5/5/5/5/5/5 and 5/5/5/5/5/4 replicates in 2 experiments. ANOVA followed by t-tests were used for statistical analysis. (D) Culturing bone marrow cells and counting the various CFUs in the presence of EDA compared to pFN showed a decrease of progenitor-CFUs (GEMM) and increase in CFUs along the myeloid lineage (GM: granulocyte/monocyte, G: granulocyte, M: monocyte), *n* = 7/7 in three experiments. Paired *t* tests were performed for all individual CFU-f populations and the total number. For statistical relationship of CT and cKO bone marrow without additives, refer to Fig 2E. Underlying data for B and C are provided in S1 Data.

Because FN production by the osteoblasts affected myelopoiesis, we aimed to determine which isoform of FN mediates this effect. We had shown previously that osteoblasts produce isoforms of FN containing EDA and/or EDB [14]. This raised the possibility that either one of the isoforms might be responsible. To purify these isoforms, we deleted FN in a cancer cell line using intron-specific *sh*RNA and followed this by stably transfecting a construct for FN that contains the EDA, the EDB, or neither domain (FN lacking both domains represents circulating plasma FN [pFN]). After single clone selection, conditioned media were collected, isoforms were purified, and the concentration and identity were determined by ELISA and western blotting (Fig 5A).

Depleting the bone marrow of CD11b^+^-cells (using antibody-coated magnetic Dynabeads) and differentiating the remaining cells with the control pFN, EDA-containing FN, or EDB-containing FN revealed that only EDA-FN increased myeloid cells (Fig 5B). In this setting too, we chose to evaluate the differentiation of HSPCs as defined by LSK and the population enriched with progenitor cells as defined by LKS^-^ (Fig 5C). EDA-containing FN enhanced the differentiation of both CT and cKO LSK and LKS^-^ cells more than the other two isoforms pFN and EDB. Of note was the higher increase in CD11b^+^-cells in the presence of osteoblasts than with the addition of the isoforms only in line with a larger role of osteoblasts in supporting hematopoiesis (Figs 4C and 5C). In addition, while EDA only increased CD11b^+^-cells differentiation from LSK, it was able to increase both CD11b^+^- and CD11b^+^Gr1^+^-cell differentiation from the LKS^-^ population, which contains more differentiated cells (Fig 5C). EDA-FN also affected the various CFUs (Fig 5D). These changes are opposite to the changes seen in cKO (and, hence, absence of EDA) compared to CT (shown in Fig 2E).

Thus, EDA-FN enhances myelopoiesis.

### EDA-Containing FN Raises CD11b^+^-Cells by Acting on Integrin-α5β1

**Fig 6:**
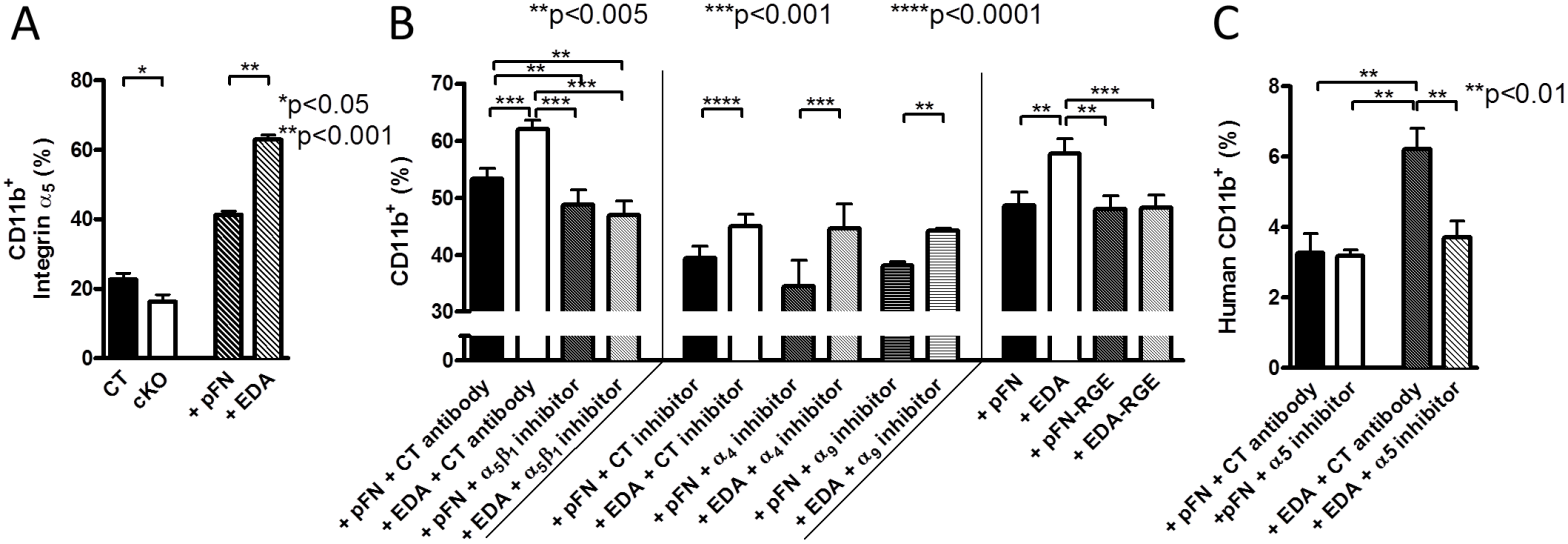
α5β1 integrin mediates EDA effects. (A) α5 integrin expression on CD11b^+^-cells in cKO (compared to CT) was opposite to that in cells treated with EDA-FN (compared to pFN), *n* = 11/9/7/7 in three experiments. Expression was evaluated by flow cytometry. Student’s *t* tests were used for statistical evaluation. (B) Inhibition of α5β1 using an antibody prevented EDA-mediated increase in CD11b^+^ differentiation (left), whereas inhibiting other integrins (α4 or α9) had no effect (middle) compared to the CT inhibitor. EDA effects are mediated by the presence of an RGD sequence in the molecule, because EDA-FN that contains a single mutation in the RGD sequence was no longer able to increase CD11b^+^-cells (right), *n* = 18/18/8/7/6/6/18/18/5/5/6/6/6/6 in four experiments, except the last four treatments, which were performed in two experiments. (C) EDA-FN-mediated stimulation of myeloid differentiation was not limited to murine cells but also seen with human cells and could be inhibited with an antibody directed against α5, *n* = 8/4/8/8 in two experiments. ANOVA followed by *t* tests for comparisons between the individual treatments was used for statistical analysis in B and C. Underlying data for A–C are provided in S1 Data.

The EDA domain binds to α4β1 and α9β1 [21], and its presence in FN enhances binding to α5β1 [22]. We therefore asked which receptor is involved. The expression profile of these integrins on CD11b^+^-cells in cKO and in response to EDA-FN was evaluated. Only α5 was significantly affected in opposite directions (lower in cKO versus CT, presumably because of the absence of EDA, and higher when EDA-FN was added compared pFN addition) (Fig 6A). In line with these findings, inhibiting α5β1 prevented the stimulatory effect of EDA-FN on CD11b^+^-cell percentages, while inhibiting neither α4 nor α9 integrin prevented the increase in CD11b^+^-cells in the presence of EDA-FN (Fig 6B). EDA-containing FN effects were mediated by binding of the RGD-sequence to α5β1 (in the presence of the EDA domain), because using two constructs—pFN and EDA-FN containing a mutated RGD-to-arginine-glycine-glutamic acid (RGE) sequence unable to bind to α5β1—showed that EDA-RGE no longer enhanced CD11b^+^-cells (Fig 6B).

Using human CD34^+^ stem cells, we confirmed that EDA-FN boosted the percentage of CD11b^+^-cells in culture and that inhibiting α5 prevented this effect (Fig 6C). This shows that EDA-containing FN increases CD11b^+^-cells by acting on α5β1 integrin.

### Loss of Osteoblast-Derived FN Diminishes Cancer Growth

Next, we asked what the in vivo implications for the changes in myelopoiesis are. In particular, the lowered CD11b^+^Gr1^+^, mMDSCs, and gMDSCs raised the possibility that processes normally attributed to MDSCs might be affected. Classically, MDSCs are detrimental to the immune response towards cancer, and, hence, a reduction in the numbers and/or function of MDSC-like cells would diminish cancer growth [35].

**Fig 7:**
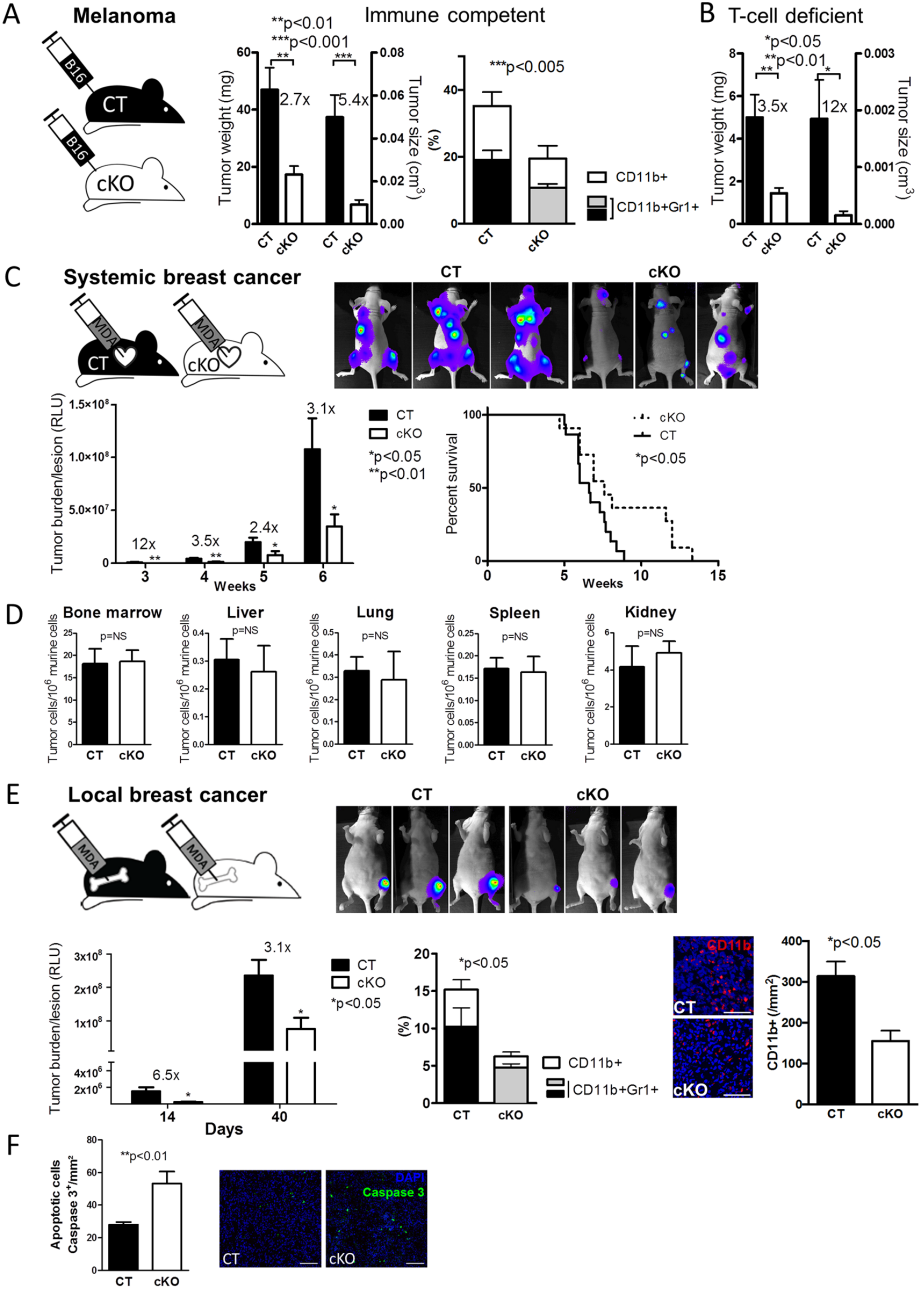
**Loss of osteoblast-FN diminishes melanoma cancer growth.** (A) Melanoma B16 subcutaneous tumors showed delayed growth in cKO animals compared to CT. The fold increase in tumor growth of CT versus cKO is shown above the respective columns. The percentage of CD11b^+^- and CD11b^+^Gr1^+^-cells was diminished in tumors induced in cKO mice, *n* = 15/15 in five experiments for tumor growth and *n* = 12/10 in four experiments for flow cytometry. Student’s *t* tests were used for statistical analysis between CT and cKO. **The role of T-cells.** (B) Similarly, melanoma B16 subcutaneous tumors showed delayed growth in cKO animals lacking thymus-derived T-cells compared to T-cell–deficient CT, *n* = 6/6/6/6 in three experiments. Student’s *t* tests were used for statistical comparisons between CT and cKO. The fold increase in tumor growth of CT versus cKO is shown above the respective columns. Tumor growth was less than in immune competent mice, shown in panel C, because of the different duration of the experiment: 14 d in immune competent mice and 4 d in T-cell–deficient mice. **Confirmation in breast cancer models.** (C) Intracardiac injection of breast cancer cells was associated with suppressed growth and prolonged survival in cKO animals, *n* = 20/20 in eight experiments for growth and *n* = 15/11 in six experiments for survival. Repeated measures ANOVA followed by Student’s *t* tests for statistical comparisons between CT and cKO were used. Kaplan–Meier method was applied for survival analysis. (D) Homing of cancer cells to the bone marrow and various organs failed to show a difference between CT and cKO animals. 10^5^ breast cancer cells were injected intracardially, and organs evaluated after 24 h for the number of cancer cells by qPCR, *n* = 22/24 for bone marrow and 6/5 for tissue. Comparisons were performed by *t* tests. (E) Intratibial injection of breast cancer cells into the bone marrow was associated with diminished growth that is more pronounced early on. Flow cytometry analysis of immune cells in the tumors showed lower percentages of CD11b^+^-, and CD11b^+^Gr1^+^-cells and histologic analysis confirmed the decrease in CD11b^+^-cells, *n* = 13/10 in five experiments for tumor growth, *n* = 10/11 for flow cytometry, and *n* = 3/3 for histological analysis. CD11b^+^-cells are shown in red (Bars represents 50 μm). (F) After 40 d, more Caspase 3^+^ apoptotic cells were found in tumors of cKO mice, *n* = 7/4 biological replicates in two experiments, bars represent 200 μm. Growth was evaluated by repeated measures ANOVA followed by Student’s *t* tests in evaluation of tumor burden; only *t* tests were used in the remaining two graphs. Underlying data for A–F are provided in S1 Data.

To test this, we injected 10^6^ cells of melanoma cancer cells (B16-F10) subcutaneously in the flank of CT and cKO immune competent mice and evaluated growth. As expected, the growth of B16 melanoma tumors in cKO animals was diminished, as were myeloid cells in the tumors (Fig 7A).

MDSCs induce regulatory T-cells that can inhibit the immune response [36–38]. To evaluate the role of T-cells in cKO mice, we induced B16 tumors in athymic mice carrying a *foxn*-mutation, leading to failed T-cells maturation. Despite the absence of thymus-schooled T-cells in the mice, suppressed cancer growth was confirmed in cKO (Fig 7B). It should be noted, however, that cancer growth is accelerated in these mice, and, therefore, cancer weight and size were evaluated after 14 d in immune competent mice (Fig 7A) and after 4 d in the athymic (immune deficient) mice (Fig 7B).

This suggests that thymus-derived T-cells are not a prerequisite for the in vivo effects caused by CD11b^+^-cells developing in the absence of osteoblast-FN.

We next aimed to confirm reduced cancer growth in cKO using another model. A human breast cancer cell line selected to home to the bone marrow and form bone metastases (MDA-MB-231B/luc^+^) was introduced in athymic mice by intracardiac injection [16,39]. The growth of metastatic bone lesions was evaluated by bioluminescence imaging and found to be suppressed in cKO mice, resulting in prolonged survival by 15% (Fig 7C). The difference in growth was not due to a decrease in homing of cancer cells to the bone marrow or other organs (Fig 7D). We then injected the cancer cells directly into the bone marrow of the tibia and found smaller lesions in cKO animals, particularly during early stages of cancer establishment (Fig 7E). Because loss of circulating FN diminishes cancer growth [16], it is possible that part of the decrease in growth in this model is due to depletion of osteoblast FN. Flow cytometry and histology substantiated lowered CD11b^+^-cells in tumors from cKO mice. Of note was an increase in apoptosis in the tumors in cKO animals as shown by the quantification of caspase 3^+^-stained cells (Fig 7F). This confirmed decreased cancer growth in cKO animals.

### CD11b^+^-Cells Exert Direct Effects on Cancer Cells In Vitro Depending on Whether They Were Preexposed to EDA-FN

**Fig 8:**
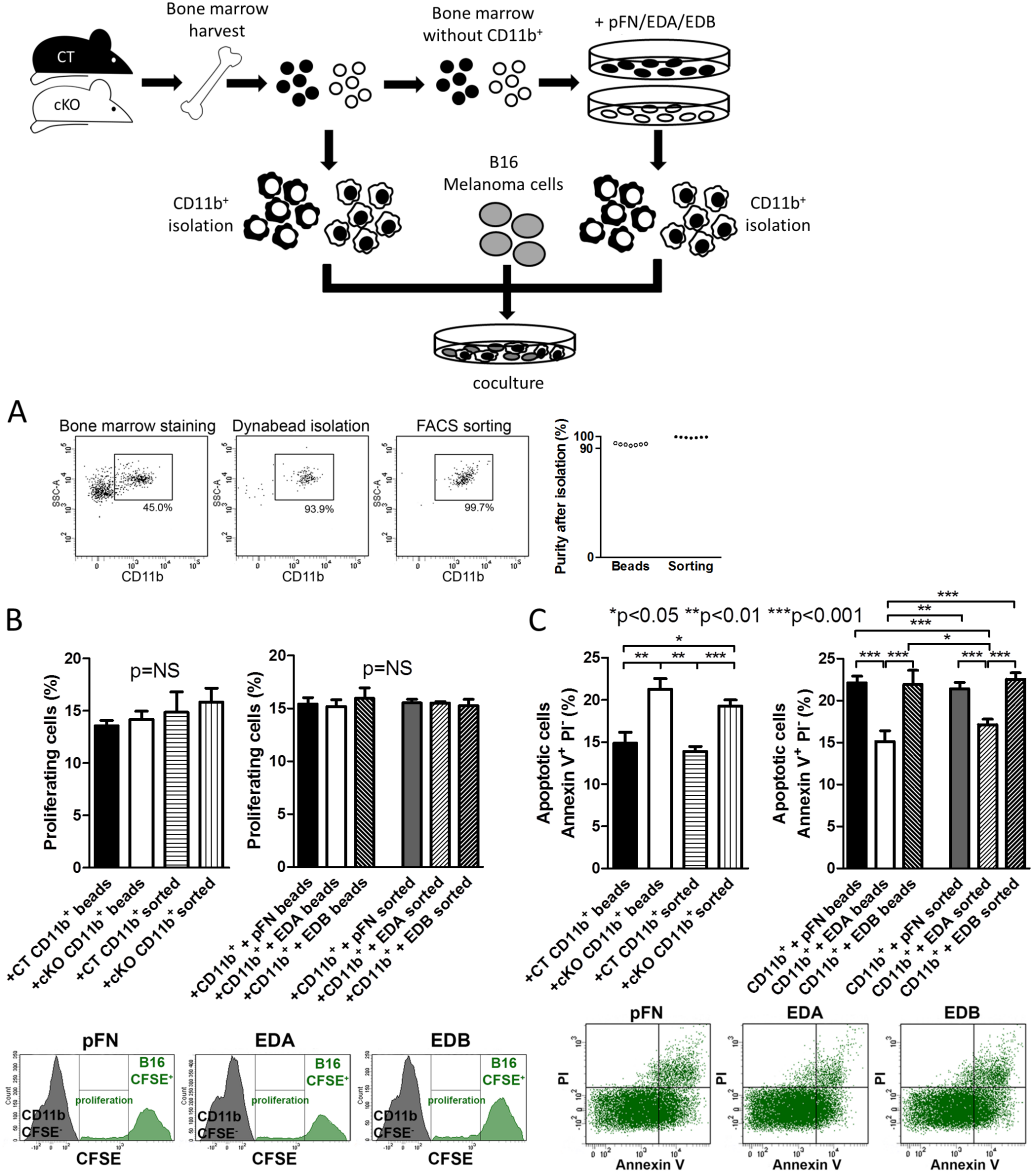
CD11b^+^-cells from cKO mice affect cancer cells in a direction opposite to EDA-FN exposure in vitro. (A) For the isolation of CD11b^+^-cells, two methods were used: isolation with antibody-coated Dynabeads and flow cytometry-based sorting. Both isolation methods achieved high purity of CD11b^+^ myeloid cells, which was evaluated by flow cytometry, *n* = 7/7. (B) Proliferation of B16 melanoma cells was not affected by coculture with freshly isolated cKO CD11b^+^-cells or CD11b^+^-cells newly formed in the presence of EDA-FN, irrespective of whether Dynabeads or flow cytometry sorting was used for isolation of CD11b^+^-cells. (C) Apoptosis was increased in B16 melanoma cells when cocultured with CD11b^+^-cells from cKO mice and decreased when cocultured with CD11b^+^-cells formed in the presence of EDA, but not when pFN or EDB were used. Cancer cell apoptosis did not differ between isolation of CD11b^+^-cells with Dynabeads or flow cytometry sorting, *n* = 14/16/7/7 for CT versus cKO and *n* = 12/12/12/9/12/9 for isoforms in three experiments. Freshly isolated CD11b^+^-cells from bone marrow were used for CT and cKO experiments. For the experiments using the isoforms, bone marrow was first depleted of CD11b^+^-cells and cultured with the isoforms (pFN, EDA, EDB). Newly formed CD11b^+^-cells were isolated after 24 h and added to the cancer cells. In all experiments, 2x10^6^ CD11b^+^ cells were cocultured with 10^6^ B16 tumor cells (at a 2:1 ratio) for 24 h. Student’s *t* tests were used for comparisons between CT and cKO and ANOVA followed by *t* tests was performed for FN-treated groups. Underlying data for A–C are provided in S1 Data.

In order to determine whether myeloid cells from cKO and those differentiated in response to EDA-FN exposure affected cancer cells directly, we aimed to isolate myeloid cells in large numbers. We therefore first tested whether there was a difference between isolation based on antibody-coated magnetic beads and cell sorting by flow cytometry. Both methods provided more than 90% purity of isolated CD11b^+^-cells (Fig 8A). We then isolated myeloid cells from CT and cKO animals and cocultured them with B16 melanoma cells using both methods. After 24 h, we found a significant increase in apoptosis of cancer cells but no change in proliferation (Fig 8B and 8C). In contrast, CD11b^+^-cells developed in the presence of EDA-FN and cocultured for 24 h with melanoma cells resulted in the opposite effect on apoptosis of melanoma cells, namely a decrease, without affecting proliferation (Fig 8B and 8C). This effect was specific for EDA-containing FN, as it did not occur in CD11b^+^-cells exposed to the other isoforms. It should be noted that the addition of the isoforms themselves on cancer cells affected neither proliferation nor apoptosis when evaluated after 24 h, but that the apoptosis was generally lower in the absence of immune cells (S4 Fig) [40,41]. There were no differences between the results obtained using bead-based isolation or sorting, which suggests that both methods can be used to evaluate the role of EDA-FN in myeloid cells.

Thus, exposure of myeloid cells to EDA-FN changes their behavior in the opposite direction to cKO and allows them to affect cancer cells in vitro.

### Suppressed Cancer Growth in cKO Animals Results from an Intrinsic Change in Myeloid Cells

**Fig 9:**
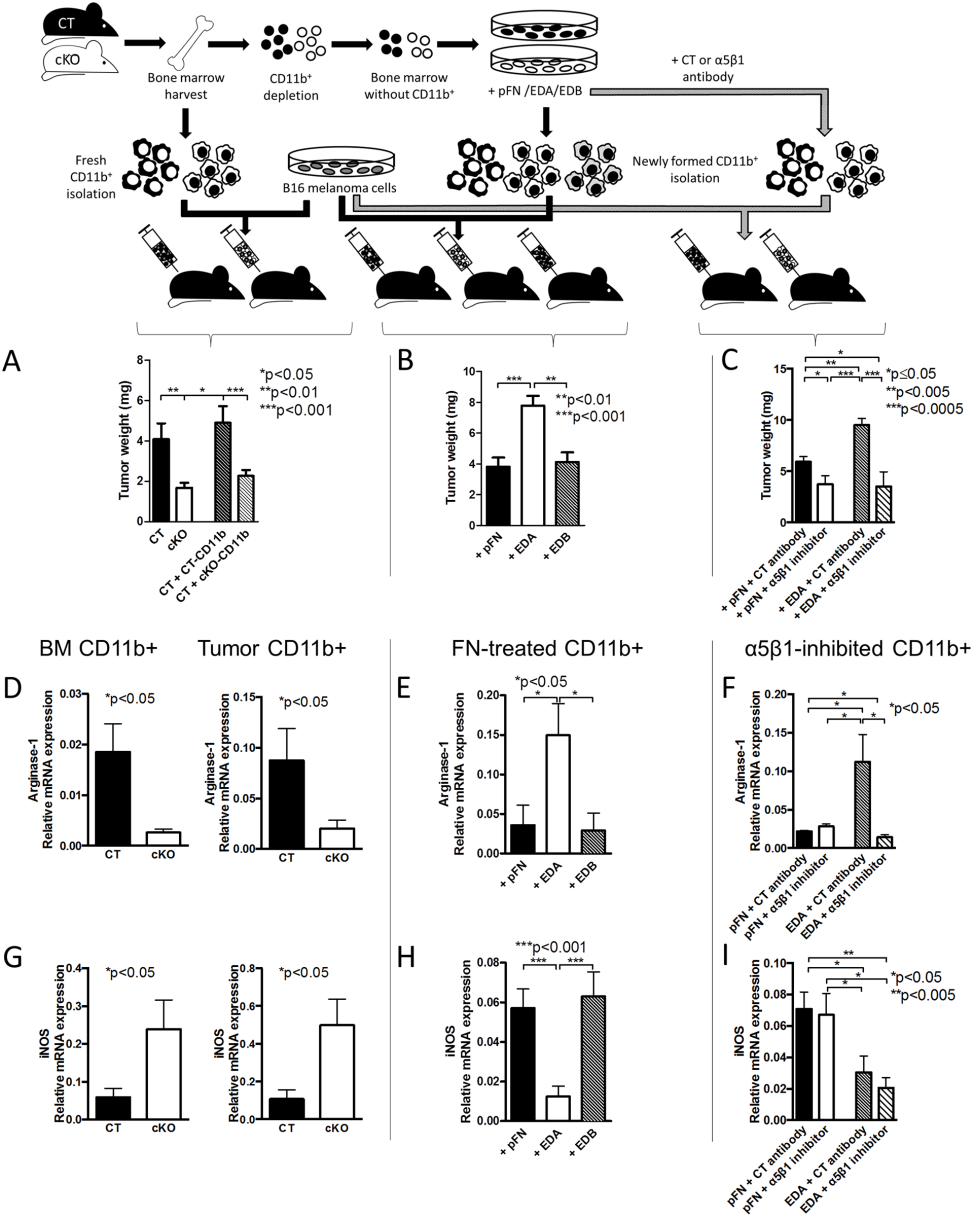
**Myeloid cells are intrinsically different based on exposure to EDA-FN.** (A) Adoptive transfer using similar numbers of isolated CD11b^+^-cells from cKO and CT animals mixed with B16 melanoma cells and injected subcutaneously showed a significant decrease in growth in the tumors that contained cKO CD11b^+^-cells, *n* = 16/10/18/18. Isolated CD11b^+^-cells were pooled for each experiment from at least three mice, and the experiment was repeated four times. ANOVA followed by *t* tests was performed. **EDA-containing FN, by acting on α5β1, modifies CD11b**^+^**-cell behavior and arginase-1 expression.** (B) Newly formed CD11b^+^-cells generated in the presence of EDA and then mixed with B16 melanoma cells and injected subcutaneously resulted in larger tumors compared to CD11b^+^-cells generated in the presence of pFN or EDB *n* = 7/7/5 in three experiments. (C) Inhibition of EDA effects using an antibody directed against α5β1 prevented growth stimulation by EDA, *n* = 6/6/6/6 in two experiments. ANOVA followed by *t* tests was used for comparisons. (D) Expression of arginase-1 in CD11b^+^-cells isolated from the bone marrow of CT and cKO animals, from the tumors in CT and cKO animals, (E) from CD11b^+^-cells generated in the presence of pFN, EDA, or EDB, or (F) from CD11b^+^-cells in which EDA effects were prevented by inhibiting integrin α5β1. EDA resulted in opposite effects to cKO or use of the inhibitory antibody. (G) Even though inducible nitric oxide synthase (iNOS) was elevated in the absence of EDA (cKO bone marrow and tumor) and (H) diminished in the presence of EDA (+EDA columns), (I) the inhibitor of α5β1 failed to prevent the effect of EDA on the mRNA expression of iNOS, suggesting that it is not involved in mediating EDA effects through α5β1 integrin, *n* = 8/10, 5/7, 13/12/8, 8/8/8/8. The numbers represent biological replicates. The samples were collected over four separate experiments for bone marrow, three experiments for tumor, three experiments for EDA treatment, and three experiments for use of the inhibitory antibody. ANOVA followed by *t* tests was performed. Underlying data for A–I are provided in S1 Data.

We next aimed to confirm that the loss of osteoblast FN affects CD11b^+^-cell behavior and not merely their numbers in vivo. Equal numbers of isolated CD11b^+^-cells from the bone marrow of CT and cKO animals (2x10^6^) were mixed with 10^6^ B16-melanoma cancer cells and injected subcutaneously in the flanks of CT mice. Cancer growth was diminished in athymic CT animals when CD11b^+^-cells were obtained from cKO animals compared to CD11b^+^-cells obtained from CT (Fig 9A). The relationship was similar to melanoma cells injected in CT and cKO animals without addition of CD11b^+^-cells (Fig 9A, left two columns).

This confirms that myeloid cells not exposed to osteoblast FN result in smaller cancer lesions due to an intrinsic change in the myeloid cells.

### Exposure of Myeloid Cells to EDA-FN Increases Cancer Growth, Whereas Inhibiting α5β1 Reduces Growth

To establish a causal relationship between EDA-FN and the immune response, we depleted CT bone marrow of CD11b^+^-cells and treated the bone marrow with pFN, EDA-FN, or EDB-FN for 24 h. Newly formed CD11b^+^-cells were isolated, mixed with B16 cells, and injected subcutaneously in athymic CT mice. After 4 d, tumor cells mixed with CD11b^+^-cells exposed to EDA-FN showed more growth than those exposed to pFN or EDB-FN even though the percentage of exogenously added CD11b^+^-cells did not differ (Fig 9B and S5 Fig).

Repeating the experiment using CT-CD11b^+^-cells differentiated in the presence of EDA-FN with and without an inhibitor of α5β1 revealed that inhibiting α5β1 for 24 h in differentiating CD11b^+^-cells was enough to suppress cancer growth (Fig 9C).

In summary, CD11b^+^-cells from cKO animals inhibit cancer growth, whereas those exposed to EDA-FN enhance cancer growth. Finally, inhibiting α5β1 during CD11b^+^-differentiation suppresses cancer growth.

### EDA-FN Changes the Expression of Immune Factors in Myeloid Cells In Vitro and In Vivo

Adoptive transfer experiments using similar numbers of CD11b^+^-cells from cKO and CT animals showed that, in the absence of FN in the osteoblasts, cancer growth is diminished (Fig 9A). In addition, using the same number of CD11b^+^-cells in similarly designed experiments either differentiated in the presence of pFN or EDA-FN revealed that exposure to EDA-FN resulted in enhanced cancer growth (Fig 9B). Thus, similar numbers of CD11b^+^-cells in the tumors resulted in opposite effects on growth in cKO-CD11b^+^-cells or EDA-exposed CD11b^+^-cells. We therefore evaluated the expression of immune factors in these different CD11b^+^-cells by measuring relative mRNA expression by quantitative reverse transcription PCR of four molecules, starting with arginase-1 and inducible nitric oxide synthase (iNOS), both of which are mediators of MDSC function [42]. We found that cKO-CD11b^+^-cells from bone marrow or tumors as well as α5β1 integrin inhibitor-treated cells showed lowered mRNA expression of the anti-inflammatory arginase-1, consistent with the inhibition of cancer growth (Fig 9D and 9F). In contrast, differentiating the depleted bone marrow in the presence of EDA-FN resulted in an opposite expression pattern in the CD11b^+^-cells (Fig 9E). In line with the reciprocal regulation of arginase-1 and iNOS [43], iNOS was increased in CD11b^+^-cells from cKO animals. Even though iNOS showed opposite changes in cKO compared to EDA-treated cells (Fig 9G and 9H), inhibition of α5β1 failed to change iNOS expression towards cKO cells (Fig 9I). This suggests that iNOS did not respond to α5β1 stimulation by EDA-FN. Interleukin-6 (IL-6) showed the same pattern as iNOS and failed to respond to α5β1 inhibition (S6 Fig). In view of the fact that α5β1 inhibition on CD11b^+^-cells reversed EDA effects on cancer growth in vivo, the changes in iNOS and IL-6 seemed irrelevant in our model. Finally, TNF-α did not change (S6 Fig).

Taken together, these data show that EDA-FN enhances arginase-1 expression of myeloid cells, possibly contributing to immune suppression in cancer.

### Arginase Mediates EDA-FN Stimulation of Cancer Growth

Because EDA-FN increases arginase-1 mRNA expression and cancer growth, while inhibition of EDA-FN effects using the integrin α5β1 inhibitor diminishes arginase-1 and cancer growth, we sought to determine whether arginase indeed mediates EDA stimulation of cancer growth.

**Fig 10:**
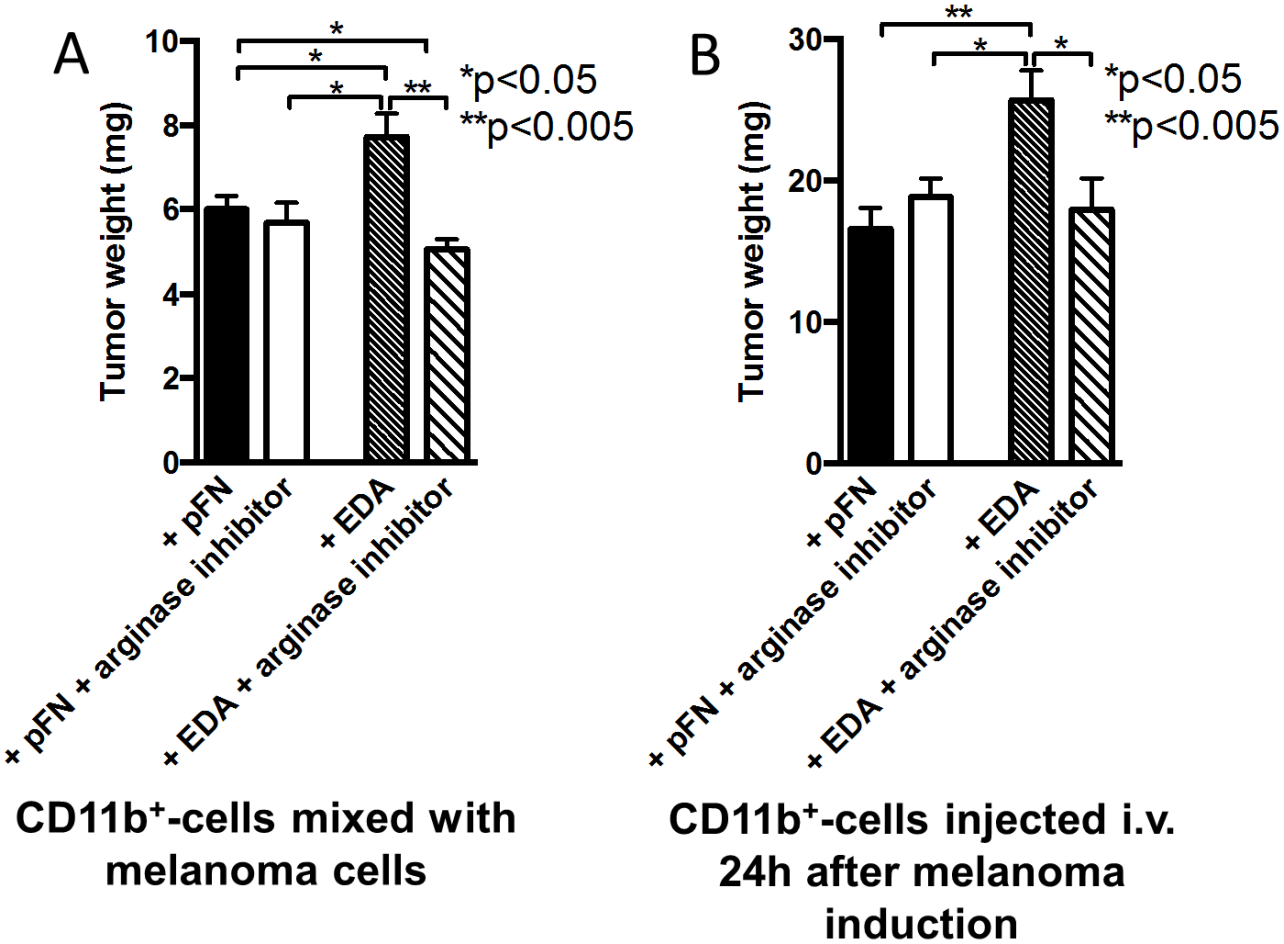
Inhibiting arginase-1 counteracts the effects of EDA-FN. (A) Adoptive transfer of newly formed CD11b^+^-cells (2x10^6^) exposed to pFN or EDA for 24 h and in the last hour to the arginase inhibitor nor-NOHA was mixed with melanoma B16 cells (10^6^) and injected subcutaneously. Cancer growth was increased with exposure to EDA, but exposure to the arginase inhibitor reversed this increase back to growth using pFN-treated cells, *n* = 4/4/5/5. (B) Newly formed CD11b^+^-cells exposed to EDA for 24 h and the arginase inhibitor during the last hour (4x10^6^) were injected intravenously 1 d after subcutaneous injection of B16 cells. The addition of the arginase inhibitor prevented enhanced cancer growth seen with EDA exposure alone. Growth was evaluated after another 3 d, *n* = 6/6/6/6. For statistical evaluation, ANOVA followed by *t* tests was performed. Underlying data for A and B are provided in S1 Data.

Two different approaches were used. In the first one, isolated wild-type CD11b^+^-cells were treated with pFN or EDA-FN for 24 h and in the last hour were exposed to the arginase inhibitor called nor-NOHA. These cells were then mixed with melanoma B16 cells and injected subcutaneously. Cancer growth was increased with exposure to EDA-FN (similarly to Fig 9B and 9C), but exposure to the arginase inhibitor for merely 1 h reversed this increase fully (Fig 10A). Inhibition of arginase in pFN-exposed CD11b^+^-cells had no effect.

In a second approach, we evaluated whether intravenous injection of CD11b^+^-cells exposed to EDA-FN for 24 h and the arginase inhibitor during the last hour affected the growth of an established tumor. Injecting 4x10^6^ CD11b^+^-cells treated with EDA-FN 1 d after subcutaneous injection of B16 cells enhanced cancer growth evaluated after an additional 3 d. In contrast, injection of CD11b^+^-cells exposed to EDA-FN for 24 h and arginase inhibitor in the last hour led to loss of enhanced growth (Fig 10B). Similarly to the first approach, the arginase inhibitor failed to modify the effect of pFN-treated CD11b^+^-cells.

Thus, EDA-FN enhances cancer growth by increasing arginase expression in CD11b^+^-cells.

### Exposure of CD11b^+^-Cells to EDA-FN Diminishes Liver Fibrosis

Although MDSCs are detrimental in cancer, resulting in enhanced cancer growth, they normally prevent the overshooting of the immune response during wound healing. Liver fibrosis represents an excessive wound healing process. The action of MDSCs can limit liver damage. Therefore, a decline in MDSC number in liver fibrosis should result in enhanced fibrosis [35].

To test whether the decrease in MDSCs in cKO mice and diminished anti-inflammatory arginase-1 expression (compare to Fig 9) was associated with increased liver fibrosis, we first had to determine whether FN deletion takes place in the liver, because we had shown that deletion of FN in the liver using the Mx-promoter leads to enhanced fibrosis due to increased transforming growth factor-β (TGF-β) [44]. We therefore first evaluated cre staining in the liver to exclude deletion of FN in the liver with the promoter we used. As shown (S7A Fig), no staining for cre recombinase could be detected, suggesting that the promoter we used is not expressed in the liver. In addition, neither total nor EDA-containing FN in the liver was diminished at baseline (S7B and S7C Fig). Finally, total TGF-β was similarly unchanged in cKO animals (S7D Fig). Thus, deletion of FN using the collagen α1(I) promoter did not affect liver cells or FN content in the liver.

**Fig 11:**
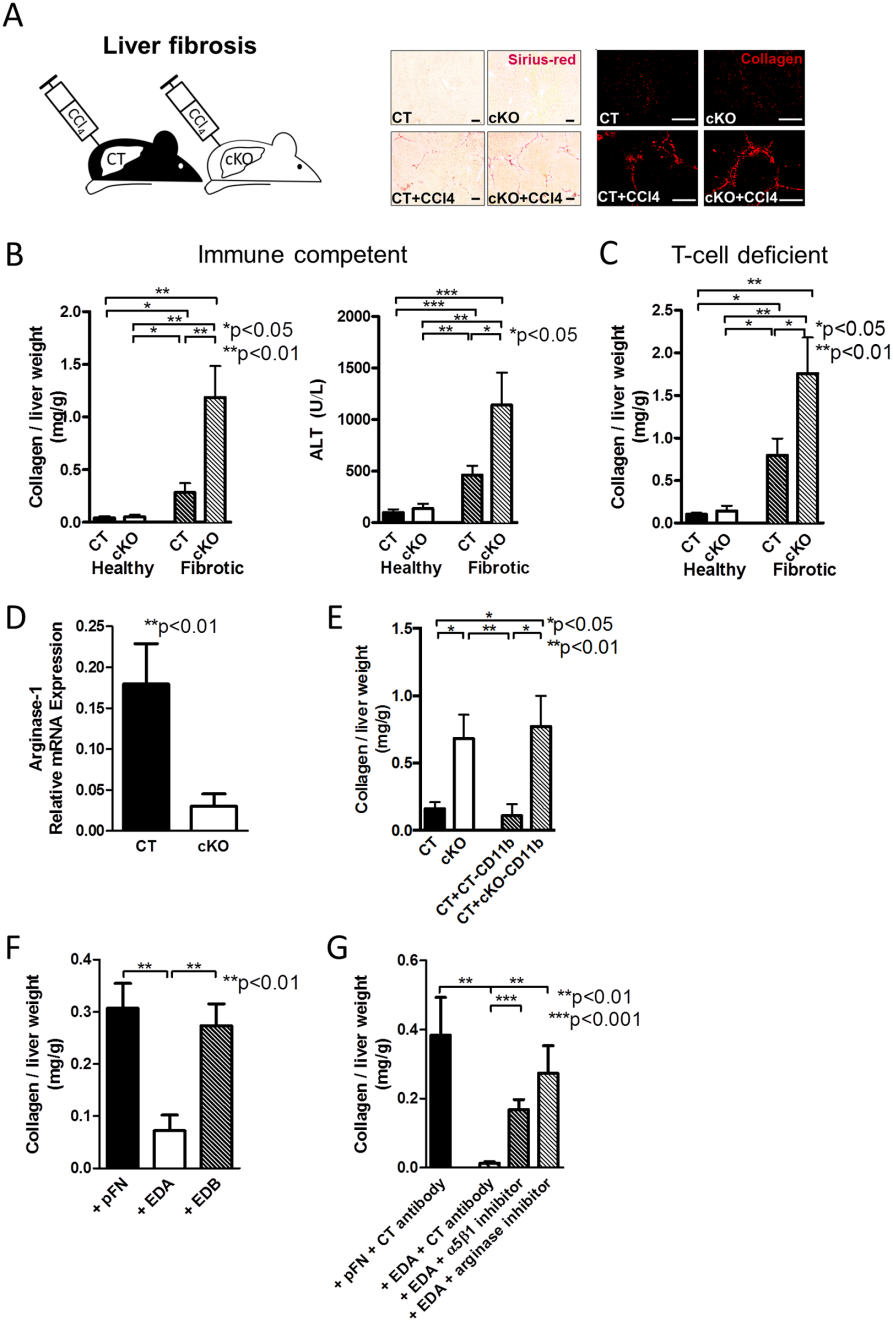
EDA-FN diminishes liver fibrosis. (A–B) Fibrosis was increased in cKO animals, as evidenced by enhanced matrix accumulation, shown using picro-sirius red staining of the matrix (A, middle), collagen I staining in red (A, right) (Bars represent 100 μm), and quantification of collagen biochemically (B, left), as well as by impaired liver function (alanine transaminase [ALT] is shown in B, right), *n* = 13/10/15/14 for collagen and 17/16/15/12 for ALT in three experiments. Fibrosis was induced by injecting CCl_4_ over 6 wk. (C) In the absence of thymus-schooled T-cells, fibrosis is still increased in cKO animals, as evidenced by collagen accumulation, *n* = 8/10/16/14 in three experiments of 6 wk exposure to CCl_4_. (D) CD11b^+^-cells isolated from the livers of healthy CT and cKO animals showed a change in arginase-1 mRNA expression similar to the changes in the myeloid cells in the bone marrow, *n* = 13/15 in four experiments. (E) The injury induced by short exposure to CCl_4_ (72 h) is increased in cKO mice or after injection of cKO myeloid cells isolated from the bone marrow 24 h after injury induction (48 h prior to euthanasia and collagen quantification), *n* = 6/6/5/5. (F) CD11b^+^-cells differentiated from the bone marrow for 24 h in the presence of EDA result in decreased matrix accumulation when injected 24 h after CCl_4_ exposure (and 48 h prior to euthanasia). This is not the case for pFN or EDB, *n* = 6/6/6. (G) In the short injury model, administration of CD11b^+^-cells exposed to an inhibitor of α5β1 integrin at the same time as EDA prevents the protective effect of EDA. An arginase inhibitor (nor-NOHA) added 1 h prior to injection of cells exposed to EDA for 24 h also prevents the beneficial effects of EDA on decreasing collagen accumulation, *n* = 4/6/6/6. ANOVA followed by *t* tests was used. Underlying data for B–G are provided in S1 Data.

We next induced liver fibrosis using the cytotoxin CCl_4_ in CT and cKO animals. After 6 wk of fibrosis induction, the amount of matrix that accumulated was enhanced in cKO, as evidenced by sirius-red staining, collagen staining, and biochemical quantification of collagen. In addition, liver injury was more pronounced in cKO animals, shown by the more pronounced elevation in alanine aminotransferase (ALT) (Fig 11A and 11B). This was not due to a change in FN content in the liver (S7B and S7C Fig) [44] and did not require the presence of thymus-schooled T-cells, because fibrosis was more pronounced in cKO animals lacking mature T-cells (Fig 11C). Analyzing CD11b^+^-cells from the livers of CT and cKO animals confirmed the decrease in arginine-1 mRNA expression as detected in the cells isolated from the bone marrow of CT and cKO animals (Figs 11D and 9D), suggesting a similar mechanism as in cancer.

In order to establish a causal relationship between the change in cytokine expression profile in CD11b^+^-cells in response to EDA and the severity of liver fibrosis, we needed to induce a large increase in matrix production in a short period. Indeed, a model was reported in which injection of the cytotoxin CCl_4_ once only was associated with a measurable increase in the amount of collagen in the liver 72 h later [45]. Injection of CCl_4_ and evaluation of the liver after 72 h confirmed an increase in collagen in CT that was more pronounced in cKO animals (Fig 11E). This suggests that cKO animals are more susceptible to matrix accumulation. Injecting CD11b^+^-cells isolated from the bone marrow of CT and cKO animals in the tail vein 24 h after a single CCl_4_ injection led to an increase in matrix accumulation in the presence of CD11b^+^-cells originating from cKO animals at 72 h (Fig 11E). Exposure of bone marrow depleted from myeloid cells to the various FN isoforms in vitro and injecting the differentiated CD11b^+^-cells after 24 h of CCl_4_ injection was associated with a decrease in matrix at 72 h when using cells exposed to EDA-FN (Fig 11F). This effect was lost in the presence of the α5β1 inhibitor, suggesting that EDA-induced change in CD11b^+^-cell behavior required the binding to α5β1 integrin (Fig 11G). Lastly, the addition of an arginase inhibitor to EDA-treated cells also prevented their protective effect, in line with the importance of arginase-1 mRNA expression in mediating EDA effects in vivo (Fig 11G).

Thus, exposure of CD11b^+^-cells to EDA-FN increases arginase-1, resulting in diminished matrix accumulation in the liver and making this mechanism beneficial in liver fibrosis.

## Discussion

**Fig 12:**
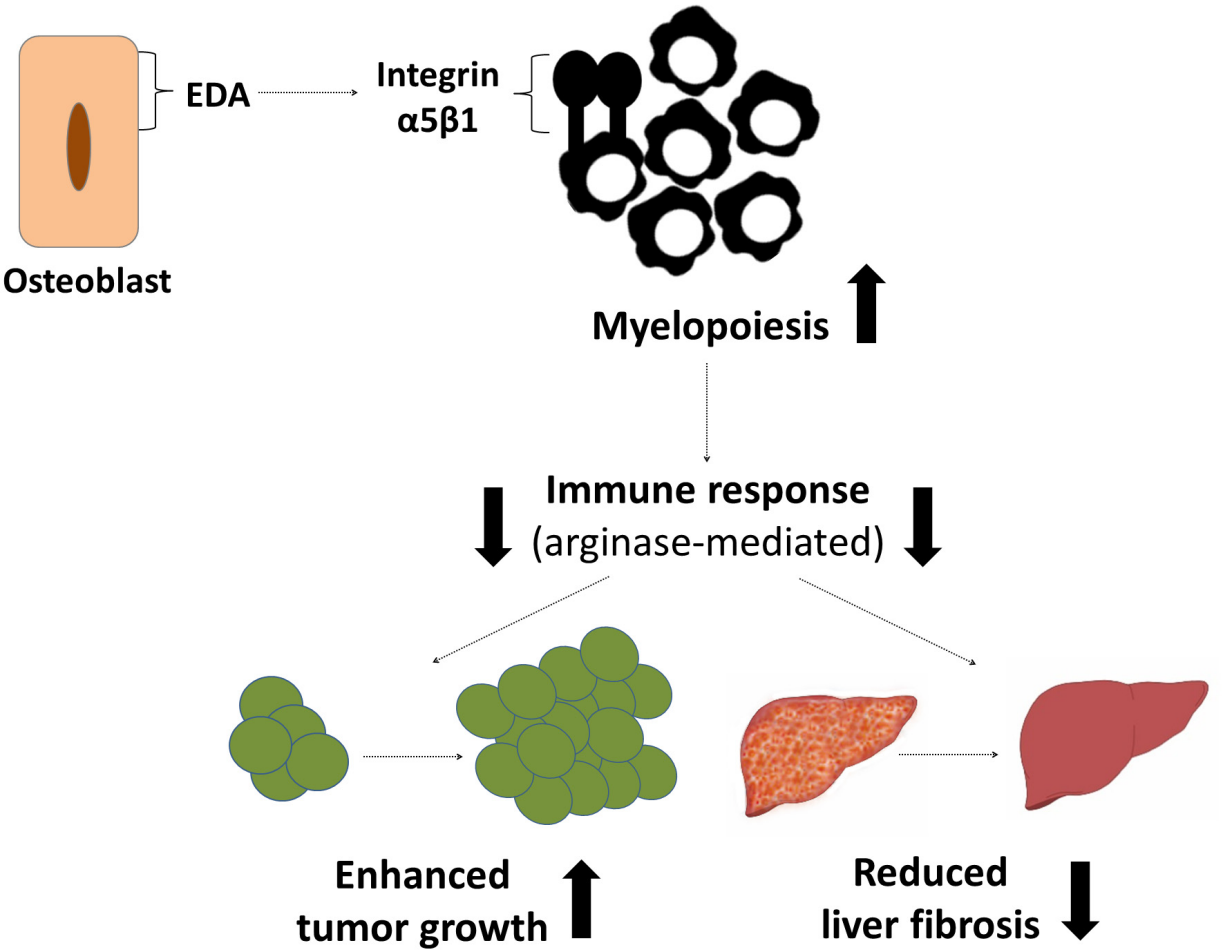
Summary. Osteoblasts produce an isoform of FN (EDA) that acts on integrin α5β1 to enhance myelopoiesis and convert the behavior of the myeloid cells into an anti-inflammatory one with increased arginase-1 expression. This mechanism is detrimental in cancer, resulting in enhanced cancer growth, but normally protects against excessive fibrotic tissue formation in liver fibrosis.

The principal finding of this study is that the FN isoform containing EDA, which is produced by osteoblasts, affects myelopoiesis and the behavior of CD11b^+^-cells by acting on α5β1 integrin and enhancing the production of the anti-inflammatory and immunosuppressive factor arginase-1. The cells exposed to EDA-containing FN are less likely to induce a strong immune response, which is detrimental in the response to cancer but beneficial in wound healing processes such as liver fibrosis (Fig 12).

Two major integrins have been identified in the bone marrow to mediate FN effects on stem cells, erythropoiesis, B-cell development, and megakaryopoiesis, namely α4β1 and α5β1 integrin [31,46–48]. An extensive role of α4β1 in hematopoiesis has been reported [19,20,28–30], but the first indication that α5β1 integrin is involved in our model was that its expression on CD11b^+^-cells changed in opposite directions in cKO bone marrow compared to CT versus EDA-treated bone marrow compared to pFN-treated bone marrow (Fig 6A). Indeed, our experimental results establish that EDA-containing FN acts on α5β1, and not on α4β1 integrin, to boost myeloid cell numbers and change their behavior (Figs 6B and 9). All FN isoforms, including the circulating isoform that lacks EDA or EDB (pFN), bind α5β1 integrins, but the presence of the EDA-domain enhances binding of the RGD-sequence to α5β1, as we and others have shown (Fig 6B and [22]). This EDA-mediated change in binding compared to pFN was sufficient to enhance myeloid differentiation (Fig 6B) and induce a different expression of the immunosuppressive arginase-1 (Fig 9E). Although the effect of EDA-FN on arginase-1 expression could be counteracted by α5β1 inhibition, this was not the case for iNOS or IL-6 (Fig 9F and 9I, S6 Fig) and could be due to the interaction of EDA-FN with α4β1 or α9β1 instead. In view of the in vivo experiments showing that pretreatment of CD11b^+^-cells with EDA-FN together with the inhibitor of α5β1 integrin or the arginase inhibitor was enough to counteract enhanced growth induced by EDA-FN exposure alone, it seems reasonable to conclude that EDA-FN acting on α5β1 integrin changes the behavior of CD11b^+^-cells towards a more suppressive phenotype characterized by increased arginase-1 and resulting in enhanced cancer growth or diminished fibrosis.

MDSCs increase CD8 T-cell apoptosis and augment the production of anti-fibrotic interferon-γ [49,50]. In line with this anti-fibrotic role of MDSCs in liver fibrosis, we found enhanced experimental fibrosis in cKO mice and treatment of myeloid cells with EDA-containing FN diminished fibrosis (Fig 11A–11C, 11F and 11G). Myeloid-derived cells interact with and act through various types of T-cells to suppress inflammation [51]. In the liver, however, neither adoptive transfer of sensitized T-lymphocytes nor depletion of T-cells affected the degree of fibrosis [52,53]. These reports thus suggest that, in liver fibrosis, T-cells are not the mediators of the inhibitory effect of MDSCs and offer an explanation for why increased fibrosis in cKO animals was maintained in the absence of thymus-schooled T-lymphocytes (Fig 11C). In contrast to liver fibrosis, published reports support a role for T-cells in mediating MDSCs’ effects in cancer [38,51,54]. MDSCs act in part through the production of arginase-1. The ensuing depletion of arginine inhibits T-cell–mediated defense against cancer [55,56]. Because expression of immunosuppressive arginase-1 in isolated CD11b^+^-cells differed depending on whether EDA-FN was present and able to bind to its receptor or not, it seemed reasonable to implicate this molecule in mediating EDA-FN effects. We show that EDA-FN increases cancer growth, and that an arginase inhibitor that does not affect nitric oxide synthase prevents growth stimulation by EDA-FN (Fig 10) despite the absence of thymus-schooled T-cells in the adoptive transfer experiments. In addition, we demonstrate that CD11b^+^-cells from cKO animals, which are not exposed to osteoblast FN in vivo, diminish cancer growth or increase liver fibrosis even in the absence of T-cells in athymic mice lacking mature T-cells (Figs 7B, 9A and 11C). Our data therefore support the conclusion that myeloid cells exposed to EDA-FN exert their immune modulatory function, either directly or by yet another immune cell both in cancer and fibrosis. This effect is mediated by arginase expression and occurs, at least in part, without an absolute requirement for thymus-derived T-cells.

The decrease in myeloid cell numbers in cKO bone marrow in the absence of osteoblast FN was not alone responsible for decreased cancer growth or enhanced fibrosis. This was important to determine, because it has been reported that using an inhibitor for CD11b decreases transmigration of CD11b^+^-cells across endothelial cells and, hence, diminishes the number of infiltrating CD11b^+^-cells in tumors, resulting in smaller cancers [57,58]. Adoptive transfer experiments unequivocally showed that similar numbers of CD11b^+^-cells affected growth differently depending on whether they were exposed to EDA-containing FN or not, and whether the effects of EDA-FN were prevented. Although CD11b^+^-cells exposed to EDA-FN enhanced cancer growth, use of cKO CD11b^+^-cells or cells treated with the α5β1 inhibitor or the arginase inhibitor together with EDA-FN showed diminished or normal growth (Figs 9B, 9C and 10). It is furthermore intriguing that injection of treated CD11b^+^-cells intravenously and after establishment of the cancer or after induction of liver injury yielded a measurable effect on cancer growth and matrix accumulation depending on the molecules to which they were exposed (as shown in Figs 10B or 11G). Thus, exposure to EDA or inhibiting its effect ex vivo for merely 24 h is enough to change the future behavior of these cells in vivo.

The role of EDA-containing FN described here suggests that interfering with the action of this molecule might be beneficial in the fight against cancer. In particular, a wide variety of cancers do indeed express EDA-FN [59], and conjugates are being increasingly evaluated to bind to the various isoforms of FN and modify their actions, as was recently reported for EDB-FN [60]. Other strategies might include inhibiting the mediating receptor α5β1, which also affects neovascularization. Indeed, a humanized antibody directed against α5β1 integrin has already undergone phase I trials after reducing cancer growth in mice [61]. Our data suggest that the action of such an inhibitor goes beyond that of an angiogenesis inhibitor and makes it a more promising candidate. Lastly, because high levels of arginase expression are associated with immune evasion, the use of arginase inhibitors as modifiers of the immune response towards cancer, which we show in vivo (Fig 10B), also offers promise in the treatment of cancer [43,62].

Thus, these new findings highlight the complexity of the interaction between the skeletal and hematopoietic systems and suggest that modifying this interaction could be used to enhance the pro-inflammatory response against cancer.

## Materials and Methods

### Mice

Mice possessing a construct of the 2.3 kb proximal region of the collagen α1(I) promoter driving cre recombinase expression (Col-cre) were used to deplete FN in osteoblasts [14]. Immune competent mice were C57BL/6. Immune deficient mice were homozygote for the foxn mutation (Charles River) and mated such that the mice generated were homozygote for floxed FN, carried the collagen α1(I) promoter attached to cre, and selected for albino skin to allow for systemic bioluminescence imaging.

For subcutaneous injection, mice were anesthetized with ketamin (120 mg/kg weight) and xylazin (16 mg/kg weight). B16-F10 cancer cells (10^6^ per 300 μl PBS) were injected subcutaneously in the left flank. After 4 d in immune deficient mice or 2 wk in immune sufficient mice, tumor weight and size were determined.

Liver fibrosis was induced by injecting carbon tetrachloride (CCl_4_) (Sigma-Aldrich) using a 1:5 mixture in olive oil 3x/wk for 42 d [44]. For the short liver injury model, in order to assess effects of transfer of myeloid cells, CCl_4_ was injected once using a 1:5 mixture and the mice euthanized after 72 h.

Intracardiac injection was performed as described [63]. Briefly, mice were anesthetized (Ketamine 120 mg/kg/xylazine 16 mg/kg). A cancer cell suspension of MDA-MB-231/luc^+^ selected to home to the bone marrow and establish bone metastases (10^5^/100 μl PBS) was used [16]. Homing of tumor cells was evaluated after 24 h of cancer cell injection in bone marrow and other organs using qPCR. DNA was isolated using PeqGold TriFast (VWR) according to manufacturer’s protocol. The human MDA tumor cells were quantified using primers for the geneticin resistance gene (5ʹ-atgcctgcttgccgaata; 3ʹ-ccacagtcgatgaatccaga; Roche probe #31) and normalized to the murine housekeeping gene β-actin (5ʹ-ctaaggccaaccgtgaaaag; 3ʹ-accagaggcatacagggaca; Roche probe #64). External standard curves obtained from murine bone marrow and tumor cells mixed with bone marrow were used to quantify the absolute number of tumor cells. Tumor growth was evaluated weekly starting 3 wk after intracardiac injection by bioluminescence reporter imaging (BLI) until death or euthanasia.

Intratibial injection was performed as described [16]. Briefly, mice were anesthetized, and two holes were drilled in the left tibia. Bone marrow was flushed out from the upper hole, which was then sealed using bone wax. Cancer cells (5 x 10^4^ per 5 μl of PBS) were injected in the lower hole using a Hamilton pipette (75RN;31/2″/3S), the hole was sealed, and the skin was sutured. Tumor growth was monitored starting day 7 and up to 40 d by BLI. Tumors obtained at day 40 were used for histology, protein, and mRNA tests.

BLI was performed using IVIS-100 (Perkin-Elmer) 5 min after d-luciferin (150 mg/kg) (Synchem) injection. Lytic lesions were detected by radiography using a Faxitron. Lytic lesions on X-rays were analyzed using Image J (Wayne Rasband, NIH, Bethesda, MD).

For bone marrow transplantation experiments (BMT), mice were irradiated two times within 3 h with a total of 9.6 gray γ-irradiation. Donor mice were euthanized and bone marrow flushed. Following lysis of red blood cells, the irradiated recipient mice were injected with bone marrow cells (10^6^ per 100 μl in PBS) in the lateral vein of the tail. Two to three mice were irradiated but did not receive BMT. Leukopenia in all mice and death of irradiated non-transplanted mice within 2 wk was a criterion for successful irradiation (S3 Fig). Both CT and cKO mice were injected with bone marrow cells from CT and cKO, resulting in four groups (Fig 4A). The transplanted mice were euthanized after 30 d and bone marrow was analyzed by flow cytometry based on completion of myelopoiesis by 4 wk [64].

Adoptive transfer experiments were performed in immune deficient mice lacking mature T-cells (nu/nu). CD11b^+^-cells (2 x 10^6^) were isolated using magnetic rat IgG beads coated with an antibody directed against CD11b and later separated from the beads as described below. The isolated CD11b^+^-cells were combined with murine melanoma B16-F10 cells (1 x 10^6^) in 300μl PBS and injected subcutaneously in the left flank of immune deficient mice. After 4 d, tumor weight and size were evaluated. For the experiment in which CD11b^+^-cells were injected intravenously, B16-F10 cells were injected as described above. After 24 h, 4 x 10^6^ CD11b^+^-cells were injected intravenously into tumor-bearing mice. For the injection of CD11b^+^-cells in the short-term liver injury model (see above), CCl_4_ was injected once and 3x10^6^ CD11b^+^-cells were injected intravenously after 24 h.

The CD11b^+^-cells for adoptive transfer experiments were either isolated freshly from bone marrow of CT or cKO mice or, alternatively, CD11b^+^-depleted bone marrow was cultured with various substances, and the newly formed CD11b^+^-cells were isolated.

Mice possessing an inducible Mx promoter driving cre-recombinase expression were crossed with mice carrying loxP-flanked (floxed) FN (Mx cKO). Mx was induced at 4 wk of age using 250 μg polyinosinic-polycytidylic acid (pIpC) injected three times over 1 wk as described [44].

All in vivo studies were replicated in at least two separate experiments with similar numbers of mice in the groups in each experiment when possible. The number of replicates per group in the figure legends refers to the total number of mice evaluated. Animals were 5–9 wk old at the start of the experiments. The number of mice in the experimental groups was estimated based on the predicted effect size. This study was carried out in strict accordance with regulations in Germany regarding the use of laboratory animals. The protocols were approved by the appropriate regulatory body of the State of Baden-Württemberg in Germany (Approval Numbers are: G-48/08, G-120/11, G-182/14, G-245/14, G-263/12, G-255/14, and G-205/15). All interventions were performed under anesthesia, except i.p. and s.c. injections. Pain after intratibial injection was managed based on the approved schedules in the respective applications. Animals were monitored daily, except after intratibial injection, for which monitoring took place initially after 4–6 h, followed by every 12 h for 3 d, and then daily. Weighing was performed weekly unless concerns arose, in which case it was replaced by daily weighing. The following criteria were used for euthanasia: weight loss more than 15%, evidence of any type or degree of paralysis, decreased mobility or activity, skin ulceration over the lesion, lesions larger than 0.5 cm^3^ for melanoma s.c. or 0.8 cm^3^ for breast cancer lesions. Because liver fibrosis can be associated with ascites that may mask the degree of weight loss, we used the development of ascites or 10% weight loss as a criterion for euthanasia. All mice were killed by ketamine/xylazine anesthesia followed by terminal bleeding. All efforts were made to minimize suffering.

### Human Cells

CD34^+^ cells were obtained from Cytotech and represent cells that were being discarded because they were no longer needed. The protocol was approved by the human investigation committee at the University of Heidelberg (S-281/2014).

### Staining Protocols and Immunohistochemistry

Cryosections of 3.7% neutral-buffered formalin-fixed tumors were stained with the following antibodies: CD45 rat anti mouse 1:50 (BD Pharmingen; clone: 30F11); CD11b rat anti mouse 1:50 (BD Pharmingen; clone: M1/70); and F4/80 rat anti mouse 1:50 (eBioscience; clone: BM8). The secondary antibody used was goat anti rat Alexa 594 1:500 (Dianova #112-585-062). Active caspase 3 rabbit anti mouse 1:100 (Abcam, #ab2303) combined with goat anti rabbit Cy2 1:500 (Dianova, #111-227-003) was used to determine apoptosis in tumors.

To determine the degree of liver fibrosis, 5 μm frozen sections were stained. Sections were fixed in 4% paraformaldehyde. Extracellular matrix was stained using picrosirius red. Collagen type I was stained using rabbit anti collagen-type-I (Millipore, #AB765P); Cre-recombinase was stained using rabbit anti cre (Novagen, #69050–3) and goat anti-rabbit conjugated with Cy2 (Dianova, #111-227-003).

Sections were photographed using a Keyence Biozero microscope (Keyence, Germany) and processed using ImageJ. Quantification was performed in at least three sections per mouse in at least three mice per group or more, as noted in the figure legends. At least 0.9 mm^2^ was examined per section.

### BMD and Histomorphometry

Bone mineral density (BMD) was measured using peripheral quantitative computer tomography (pQCT) with an XCT Research SA machine (Stratech Medizintechnik, Pforzheim, Germany).

For histomorphometry, bones were fixed in 70% ethanol, embedded in polymethylmethacrylate, sectioned, and stained per Masson Goldner with hematoxilin (Gill II, Roth), acid fuchsin-ponceau xylidine, and phosphomolybdic acid-orange G and light green. For dynamic histomorphometry, calcein was administered twice at 30 mg/kg (Sigma-Aldrich). Primary cancellous bone was defined as the 120 μm band below the growth plate. Cancellous bone was defined as the remaining trabecular area that extends down 2 mm. The same sections were used for dynamic and static histomorphometry, and data obtained from evaluation of the cancellous bone area defined above are presented. The ASBMR nomenclature was used. The following measurements are mentioned: bone surface (BS), bone formation rate (BFR = MS*MAR/BS, mm^2^/mm/yr.), number of osteoclasts (Oc.N), and osteoclast surface (Oc.S). ImageJ was used (Wayne Rasband, NIH).

Sections stained with Masson-Goldner were also used for determining adipocyte number and sinusoid area.

### Cell Isolation and Culture

Osteoblasts were isolated from the calvariae of newborn mice as described [65]. Stem cell analyses in culture were performed with the MethoCult GF M3434 Assay (Stemcell Technologies). It is optimized for the detection and quantification of mouse hematopoietic progenitors in bone marrow. Also, FN isoforms were added (200 ng/ml). Quantification of the CFUs was performed using light microscopy.

MDA-MB-231B/luc^+^ was cultured in Dulbecco's modified Eagle's medium (DMEM)/10% fetal calf serum (FCS) with 800 μg/ml geneticin (Applichem) [16]. Murine melanoma B16-F10 and human CD34^+^ cells also were cultured in DMEM/10% FCS. Cells were counted using an automated cell counter (CASY-TT; Innovatis, Mannheim, Germany). MDA-MB-231B/luc^+^ were first transfected with FN 5ʹ UTR *sh*RNA in an expression vector (pLenti6/BLOCK- iT-DEST) with resistance against blasticidin S, followed by stable transfection with the pcDNA3.1/hygro (-) expression vector (Life Technologies) containing the cDNA for pFN, EDA, or EDB, with resistance against hygromycin B, and cultured in DMEM/10% FCS with 800 μg/ml geneticin, 6 μg/ml hygromycin B (both from Applichem), and 20 μg/ml blasticidin (Invivogen). To get recombinant pFN, EDA, and EDB, the cells were cultured for 1 wk without FCS, and conditioned media were collected and concentrated by ultracentrifugation (Macrosep Advance Centrifugal Devices, Pall Corporation) followed by isolation using gelatin-sepharose beads [66]. The identity of the FN isoforms was confirmed by western blotting and ELISA, and the concentration was determined by ELISA.

Hematopoietic stem and progenitor cells, used for coculturing with osteoblasts and culturing with isoforms, were isolated by flow cytometry sorting (BD FACSAria). First, bone marrow was depleted of lineage cells (B220 clone: RA3-6B2; CD5 clone: 53–7.3; Ter119; Gr1 clone: RB6-8C5 and CD11b clone: M1/70 purified; Biolegend) using protein G Dynabeads and stained with CD117 (c-kit) rat anti mouse APC-Cy7 1:100 (Biolegend; clone 238) and sca-1 rat anti mouse Pe-Cy7 1:200 (Biolegend; clone: E13-161.7). LSK are defined as c-kit^+^ Sca-1^+^ and LKS^-^ are defined as c-kit^+^ Sca-1^-^.

CD11b^+^-cells were isolated from the bone marrow using anti rat IgG or protein G Dynabeads (Invitrogen) coated with CD11b antibody (clone M1/70; BioLegend) or flow cytometry sorting (BD FACSAria) of bone marrow stained with CD11b rat anti Alexa 700 1:1600 (Biolegend; clone: M1/70). For RNA isolation, protein G Dynabeads were used, and after CD11b^+^-cell binding, beads were treated with RNA lysis buffer. For live cell sorting, anti rat IgG Dynabeads were used and treated with 0.05% Trypsin-EDTA for 10 min to separate the vital sorted cells from the beads. In coculture experiments, bone marrow cells were depleted of CD11b^+^-cells as described above and cultured (2 x 10^6^ per 300 μl of in DMEM in 96-well plate) with FN isoforms (200 ng/ml) with α5β1 inhibiting antibody (10 μg/ml; clone BMC5; Merck) or CT antibody (10 μg/ml, MOPC1, Sigma), α4 or α9 inhibitor (1.8 or 138 nM; #BIO 5192; Tocris), or CT inhibitor, which is echistatin, known to inhibit αvβ3 integrin (2.7 nM, Tocris) for 24 h. In coculture with osteoblasts (isolated as described in [14]), freshly isolated osteoblasts were cultured for 2 d in DMEM+10%FCS and 12 h with DMEM only. Bone marrow cells, LSK, or LKS^-^ (at a ratio 2:1) were added and cultured together with the osteoblasts for 24 h.

For cocultures of CD11b^+^-cells with B16-F10 tumor cells, either freshly isolated CD11b^+^ or CD11b^+^-depleted bone marrow (see above) was cultured with pFN, EDA, or EDB (200 ng/ml) for 24 h (4 x 10^7^ in 175 cm^2^ cell culture flasks) and newly differentiated CD11b^+^- cells were isolated (see above). B16-F10 tumor cells were stained with CFSE (1:100; Biolegend) to assess cell proliferation. 1 x 10^6^ B16-F10 were cocultured with 2 x10^6^ CD11b^+^ per well (200 μl DMEM; 48-well plate) for 24 h. Cells were harvested and stained with Alexa 647 Annexin-V (1:50; Biolegend) and PI (1:1000; Biolegend) to evaluate apoptosis of tumor cells.

For adoptive transfer experiments with combined cancer cell+immune cell injections, or in which CD11b^+^-cells were injected intravenously in a tumor or short-term liver injury model (see above) with newly formed CD11b^+^-cells, bone marrow was isolated, CD11b^+^-cells were depleted (see above), and bone marrow without CD11b^+^-cells (4 x 10^7^ in 175 cm^2^ cell culture flask) was cultured for 24 h either with pFN or EDA (200ng/ml) alone, or CT or α5β1 (10 μg/ml; clone BMC5; Merck) antibody for 24 h, or the arginase inhibitor nor-NOHA (N-ω-Hydroxy-L-norarginine; 1mM/10^7^ cells; #F-3685.0050; Bachem) for 1 h at 37°C before injection [67]. Newly formed CD11b^+^-cells were isolated using CD11b antibody and anti-rat IgG Dynabeads (see above).

### Flow Cytometry

Bone marrow was flushed from femora as described above, and cells were stained with the following antibodies: B220 (CD45R) rat anti mouse Biotin or FITC 1:200 (Biolegend; clone: RA3-6B2); CD4 rat anti mouse PerCP/Cy5.5 1:400 (Biolegend; clone: RM4-5); CD5 rat anti mouse Biotin 1:200 (Biolegend; clone: 53–7.3); CD8 rat anti mouse Alexa 700 1:400 (Biolegend; clone: 53–6.7); CD11b rat anti mouse Biotin 1:400 and Alexa 700 1:1600 (Biolegend; clone: M1/70); CD11c rat anti mouse Pe-Cy7 1:200 (Biolegend: clone: B418); CD16/32 rat anti mouse PE 1:100 (Biolegend; clone: 93); CD29 Armenian hamster anti mouse PE and FITC 1:100 (Biolegend; clone: HMß1-1); CD34 rat ant mouse FITC 1:50 (BD Pharmingen; clone: RAM34); CD41 rat anti mouse APC/Cy7 1:100 (Biolegend; clone: MWReg30); CD42d rat anti mouse APC 1:50 (Biolegend; clone: 1C2); CD45 rat anti mouse APC-Cy7 1:400 (Biolegend; clone: 30-F11); CD49d rat anti mouse PE 1:100 (Biolegend; clone 9C10); CD49e rat anti mouse APC 1:100 (Biolegend; clone: 5H10-27); CD71 rat anti mouse APC/Cy7 1:100 (Biolegend; RI7217); CD117 (c-kit) rat anti mouse APC-Cy7 1:100 (Biolegend; clone 238); CD127 rat anti mouse APC 1:50 (Biolegend; clone: A7R34); Gr-1 rat anti mouse 1:400 Biotin and 1:1600 Alexa 647 (Biolegend: RB6-8C5); Integrin alpha 9 goat anti mouse PE 1:50 (R&D, polyclonal); Ly6 C rat anti mouse PerCP-Cy5.5 (BD; clone: AL-21); Ly6G rat anti mouse Alexa 647 1:400 (Biolegend; clone: 1A8); NKp46 rat anti mouse PerCP-Cy5.5 1:100 (Biolegend; clone: 29A1.4); sca-1 rat anti mouse Pe-Cy7 1:200 (Biolegend; clone: E13-161.7); Ter-119 rat anti mouse 1:200 Biotin or FITC (Biolegend; clone: TER-119); streptavidin, Pacific Orange conjugate 1:400 (Life Technologies, Darmstadt, Germany).

### RNA Analysis

RNA was isolated using TriFast (PeqLab) and reversed transcribed with a protocol using oligo(dT) primers (25 ng/μl), dNTPS (10 mM), RevertAid Reverse Transcriptase (200 U/μL, Fermentas), and RiboLock RNase Inhibitor (40 U/μL, Fermentas). qPCR results were normalized to murine HPRT. The probes used were given as follows: arginase-1 #17; iNOS #76; IL-6 #6; TNFα #102; HPRT #95 (Roche). The primers used were those suggested by Roche universal probe library.

### Protein Analysis

FN and its isoforms were isolated using gelatin-sepharose beads, quantified in cell lysates, and conditioned media by ELISA as reported [65,68], and corrected to protein content measured by BCA (Pierce) when appropriate. Briefly, plates were coated with the primary antibody (pFN: F3648, Sigma, polyclonal, 0,12 μg/ml; EDA: F6140, Sigma, clone: FN-3E2, 5 μg/ml; EDB: L19-SIP (gift from D. Neri), 4 μg/ml). The standard used was human plasma. The secondary anti-FN-HRP antibody (P0246; Dako) was used (details of the assays can be found in [68]). Western blotting for EDA (using F6140, clone FN-3E2, Sigma) or EDB (using clone BC1, gift from D. Neri) was performed to confirm the identity further.

Hydroxyproline in liver lysates was measured using a biochemical method as published. Aspartate aminotransferase was measured in serum obtained at the time of euthanasia using routine clinical chemistry [69].

Active and matrix TGF-β were determined in liver samples as published [44] using a bioassay, which only detects bioavailable TGF-β, and corrected to total protein (BCA).

### Statistical Analyses

Analyses were performed using SPSS (V20). Analysis of variance and repeated measures analysis of variance tests were used as appropriate. If global probability values were smaller than 5%, subsequent comparisons between selected group pairs were then performed using Student's *t*, Mann–Whitney, or Wilcoxon paired tests as appropriate. Every intervention in vivo was repeated at least twice in separate experiments in vivo and in vitro. Results are expressed as means ± SEM.

## Supporting Information

S1 Data Excel spreadsheet containing, in separate sheets, all underlying numerical data for panels Figs 1A–1D, 2A–2E, 3A, 3B, 4A–4C, 5B–5D, 6A–6C, 7A–7F, 8A–8C, 9A–9I, 10A, 10B and 11B–11G, S1A–S1D, S2B, S2C, S3, S4A, S4B, S5A–S5C, S6 and S7B–S7D Figs. (XLSX)

S1 Fig (A) Bone mineral density (BMD) of total bone and trabecular bone was not altered in cKO mice, as measured by peripheral quantitative computer tomography (pQCT), *n* = 8/10. (B) Osteoclast numbers (absolute or corrected to bone surface) were not affected by diminished expression of FN in osteoblasts, as determined by static bone histomorphometry, *n* = 6/6. (C) The number of adipocytes (corrected to bone marrow area) was not affected in cKO mice, *n* = 6/6. (D) Similarly, no difference could be detected in the area covered by sinusoids in cKO, *n* = 6/6. (E) Four longitudinal sections of tibiae stained using the Masson–Goldner method are shown; bars represent 500 μm. *T* tests were used to compare the two groups. Underlying data for A–D are provided in S1 Data.
(TIF)

S2 Fig (A) Flow cytometry gates applied after doublet exclusion and used for defining the hematopoietic populations in the bone marrow. (B) MDSC subpopulations gMDSCs and mMDSCs were diminished in cKO even when the marker α4 was included, *n* = 15/17. (C) Further characterization of the bone marrow and spleen revealed no differences in B-cells, CD4^+^-, CD8^+^-cells, erythrocytes, or megakaryocytes in cKO mice, *n* = 12/12 for bone marrow and *n* = 5/5 for spleen. *T* tests were used for comparisons. Underlying data for B and C are provided in S1 Data.
(TIF)

S3 Fig No difference in the recovery from leukopenia in the different groups after transplantation, *n* = 7/8/6/8. Tukey’s test was used for statistical analysis. Underlying data are provided in S1 Data.
(TIF)

S4 Fig Culturing MDA (A) or B16 (B) tumor cells for 24 h with FN isoforms (pFN, EDA, or EDB) did not affect proliferation or apoptosis in these cells, *n* = 4 replicates in all groups except B16 apoptosis: *n* = 10/8/9. ANOVA was performed for statistical analysis. Underlying data for A and B are provided in S1 Data.
(TIF)

S5 Fig To assess the proportion of exogenously added CD11b^+^-cells in adoptive transfer experiments with B16 tumor cells, CD11b^+^-cells formed during exposure to FN isoforms (pFN, EDA, or EDB) were stained with CFSE. (A) In adoptive transfer experiments of B16 subcutaneous tumors (10^6^ B16 + 2 x 10^6^ CD11b^+^-cells), no difference in the percentage of total CD11b^+^-cells in subcutaneous tumors was detected. (B) The majority of the CD11b^+^-cells in the tumors were exogenously added (CFSE^+^). (C) The percentage of CFSE^+^-cells detected in the tumors did not differ between the different treatments, *n* = 6/7/5. ANOVA was performed for statistical analysis. Underlying data for A–C are provided in S1 Data.
(TIF)

S6 Fig Characterization of cytokine expression in CD11b^+^-cells in the presence and absence of osteoblast-FN (CT versus cKO) in the bone marrow (first row), after isolation from the tumor (second row), and after exposure to EDA in comparison to the pFN isoform without (third row) or with the addition of an α5β1 inhibitor (fourth row). Cytokines were assessed using mRNA expression. Arginase-1 showed diminished mRNA expression in cKO bone marrow and tumors, but the expression could be stimulated by the addition of EDA but not pFN. Inhibition of integrin α5β1 reversed the effect of EDA (first column). iNOS and IL-6 both showed opposite expression patterns compared to arginase-1 in bone marrow, tumor, and EDA-treatment, but the EDA-effects could not be reversed by inhibiting integrin α5β1. Finally, TNFα was not influenced by the various conditions, *n* = 8/10, 5/7, 8/8, 8/8/8/8. Analysis was performed by *t* tests for the first three rows and ANOVA for the fourth row followed by *t* tests. Underlying data are provided in S1 Data.
(TIF)

S7 Fig Deletion of FN in the liver using the Mx promoter attached to cre affects the hepatocytes and the hepatic stellate cells, resulting in diminished FN and increased TGF-β and, hence, an increase at baseline in collagen production that is more pronounced after fibrosis induction [44]. In order to determine whether the collagen α1(I) promoter has any activity in the liver, we stained liver sections from cKO animals against cre and compared to CT animals and to Mx-cre animals (marked as positive control). No staining was detected in collagen α1(I)-cre-harboring cKO animals (A); bars represent 100 μm. The total FN content of the liver was not affected (B), neither was the amount of EDA-containing FN (C), *n* = 9/12 for B and C. In line with these findings, total TGF-β was unchanged between CT and cKO animals (D), *n* = 7/6. Underlying data for B–D are provided in S1 Data.
(TIF)

## Abbreviations

ALT: alanine aminotransferase
BMD: Bone mineral density
CFU: colony-forming unit
CFU-GEMM: CFU-granulocyte/erythrocyte/monocyte/megakaryocyte
CFU-GM: CFU-granulocyte/monocyte
CFU-G: CFU-granulocyte
CFU-M: CFU-monocyte
cKO: conditional knockout mice
CLP: common lymphoid progenitor
CMP: common myeloid progenitor
CT: control
EDA: extra domain A
EDB: extra domain B
FN: fibronectin
gMDSC: granulocytic MDSC
GMP: Granulocyte-monocyte-progenitor
HSPC: Hematopoietic-stem-and-progenitor-cell
IL-6: Interleukin-6
iNOS: inducible nitric oxide synthase
LKS^-^: lineage^-^c-kit^+^sca-1^-^
LSK: lineage^-^c-kit^+^sca-1^+^
MDSC: myeloid-derived suppressor cell
MEP: megakaryocyte–erythroid progenitor
mMDSC: monocytic MDSC
pFN: plasma FN
pQCT: peripheral quantitative computer tomography
RGD: arginine-glycine-aspartic acid
RGE: arginine-glycine-glutamic acid
TGF-β: transforming growth factor-β
