# Supplementary figures and images for: EDA-Fibronectin Originating from Osteoblasts Inhibits the Immune Response against Cancer
Source: PLoS Biol. 2016 Sep 21;14(9):e1002562. doi: 10.1371/journal.pbio.1002562 (PMC5031442; doi:10.1371/journal.pbio.1002562)

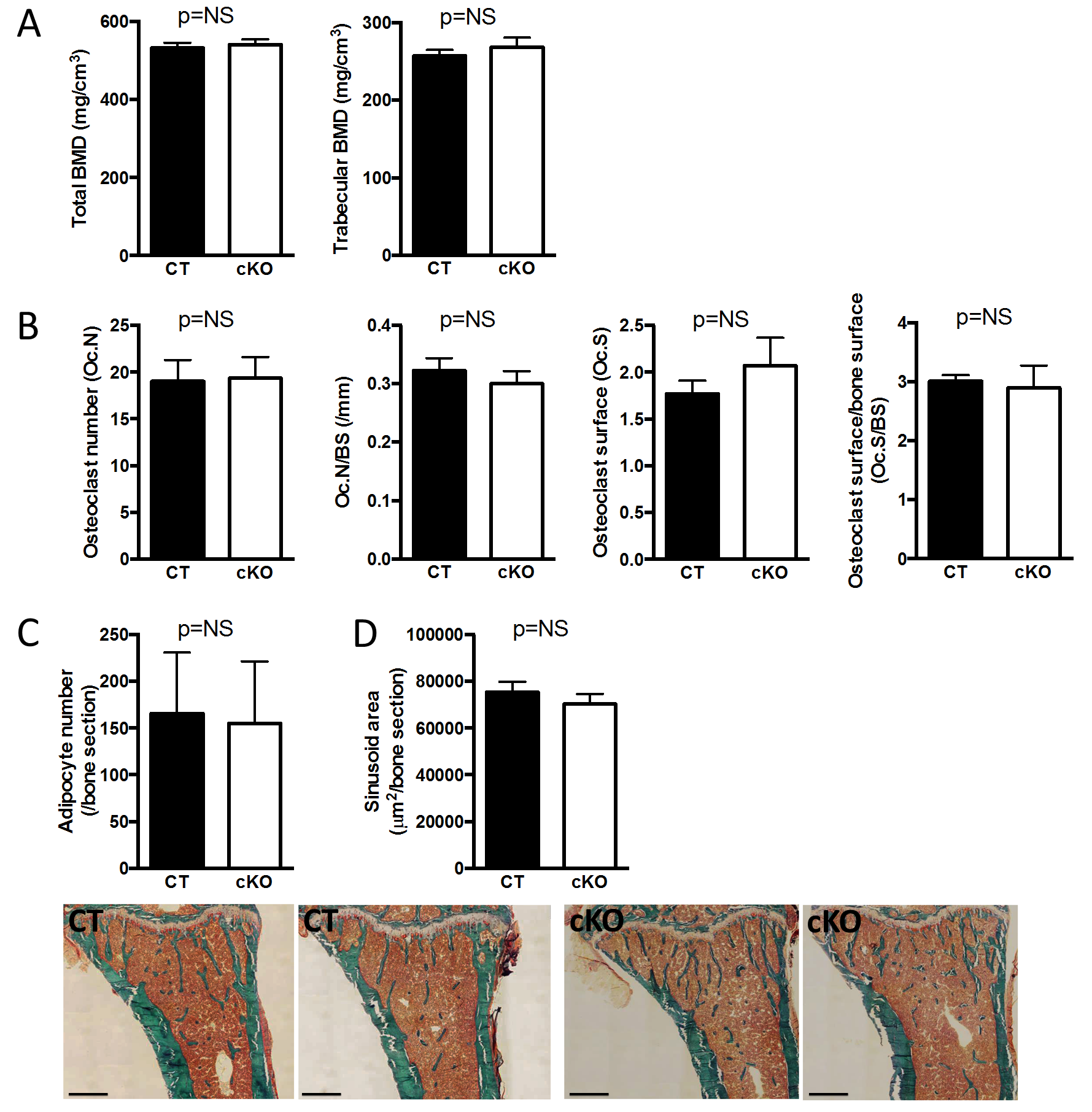

Supplement: S1 Fig — (A) Bone mineral density (BMD) of total bone and trabecular bone was not altered in cKO mice, as measured by peripheral quantitative computer tomography (pQCT), n = 8/10. (B) Osteoclast numbers (absolute or corrected to bone surface) were not affected by diminished expression of FN in osteoblasts, as determined by static bone histomorphometry, n = 6/6. (C) The number of adipocytes (corrected to bone marrow area) was not affected in cKO mice, n = 6/6. (D) Similarly, no difference could be detected in the area covered by sinusoids in cKO, n = 6/6. (E) Four longitudinal sections of tibiae stained using the Masson–Goldner method are shown; bars represent 500 μm. T tests were used to compare the two groups. Underlying data for A–D are provided in S1 Data. (TIF) [file pbio.1002562.s002.tif]

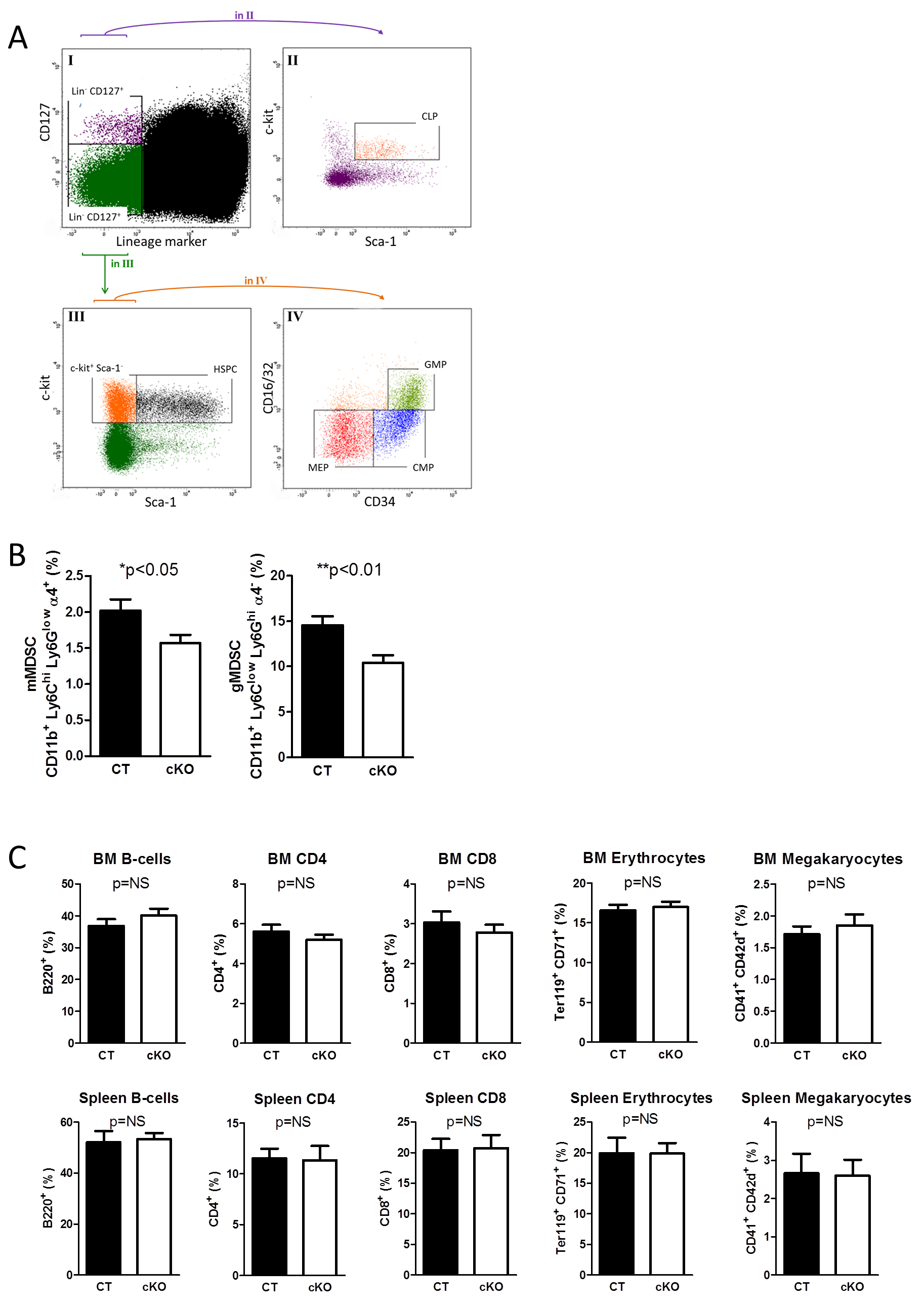

Supplement: S2 Fig — (A) Flow cytometry gates applied after doublet exclusion and used for defining the hematopoietic populations in the bone marrow. (B) MDSC subpopulations gMDSCs and mMDSCs were diminished in cKO even when the marker α4 was included, n = 15/17. (C) Further characterization of the bone marrow and spleen revealed no differences in B-cells, CD4+-, CD8+-cells, erythrocytes, or megakaryocytes in cKO mice, n = 12/12 for bone marrow and n = 5/5 for spleen. T tests were used for comparisons. Underlying data for B and C are provided in S1 Data. (TIF) [file pbio.1002562.s003.tif]

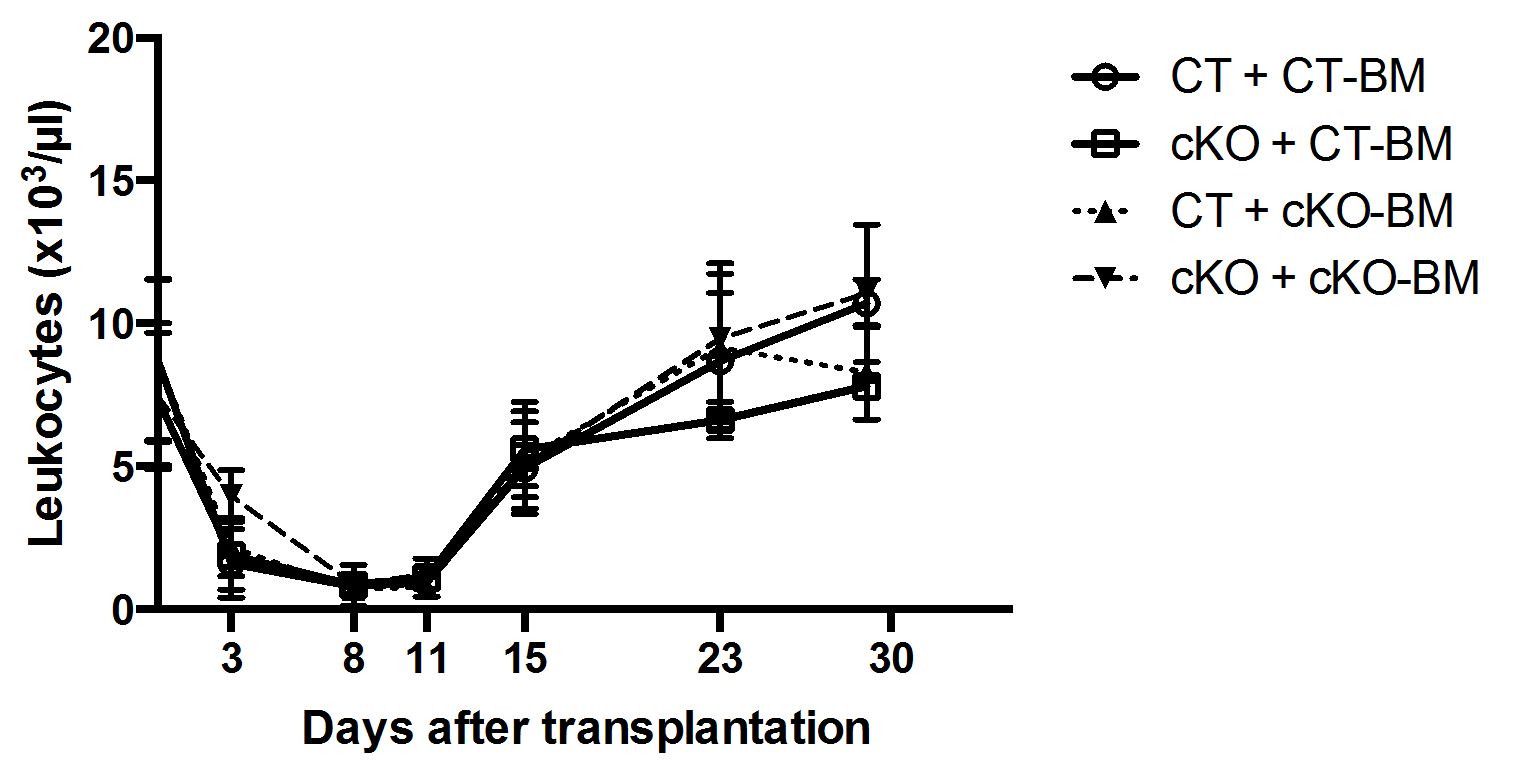

Supplement: S3 Fig — Tukey’s test was used for statistical analysis. Underlying data are provided in S1 Data. (TIF) [file pbio.1002562.s004.tif]

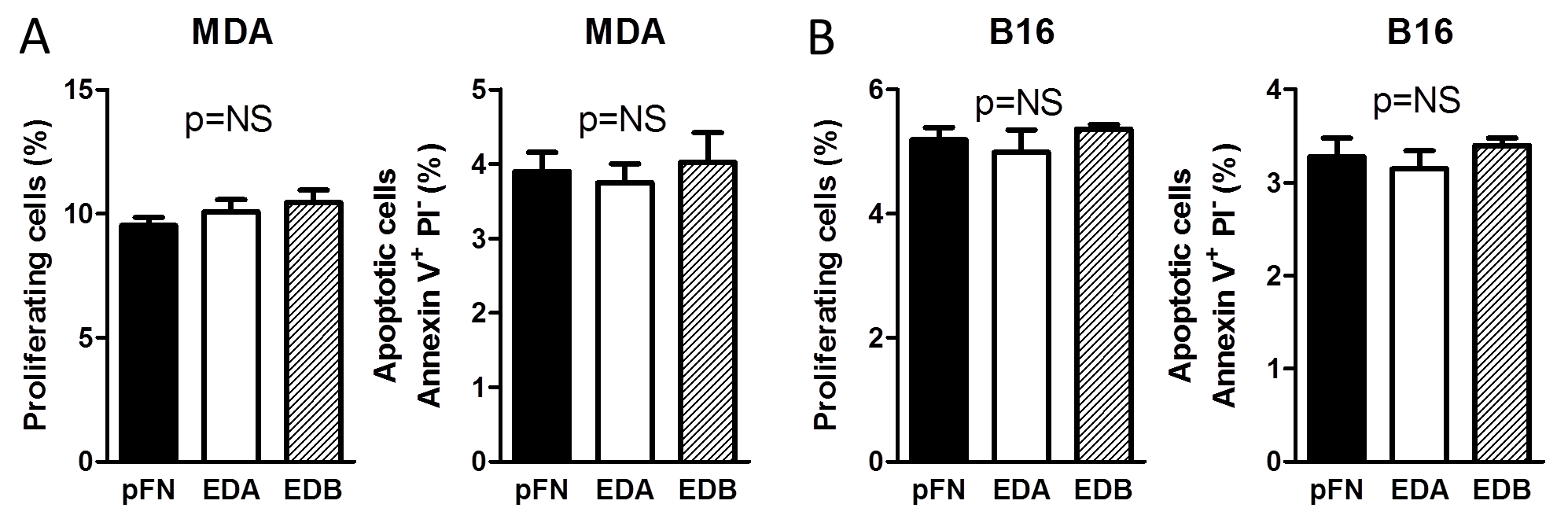

Supplement: S4 Fig — ANOVA was performed for statistical analysis. Underlying data for A and B are provided in S1 Data. (TIF) [file pbio.1002562.s005.tif]

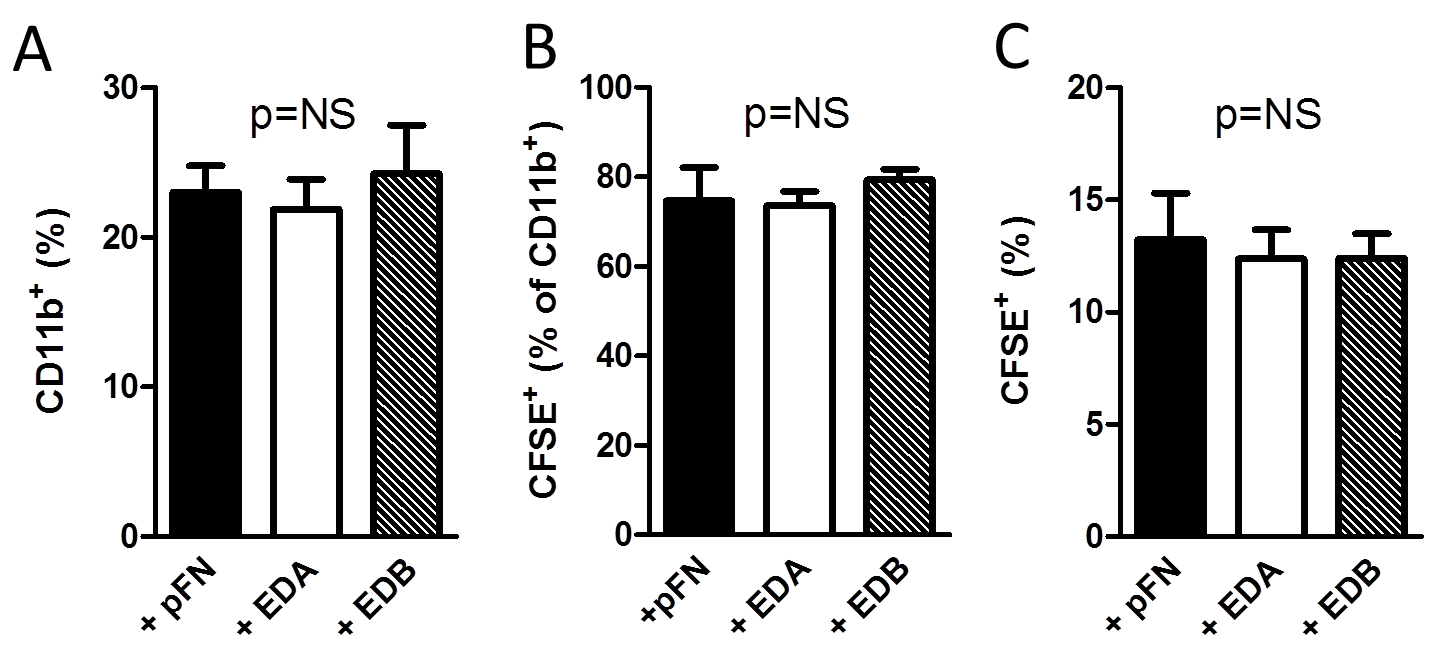

Supplement: S5 Fig — (A) In adoptive transfer experiments of B16 subcutaneous tumors (106 B16 + 2 x 10^6^ CD11b+-cells), no difference in the percentage of total CD11b+-cells in subcutaneous tumors was detected. (B) The majority of the CD11b+-cells in the tumors were exogenously added (CFSE+). (C) The percentage of CFSE+-cells detected in the tumors did not differ between the different treatments, n = 6/7/5. ANOVA was performed for statistical analysis. Underlying data for A–C are provided in S1 Data. (TIF) [file pbio.1002562.s006.tif]

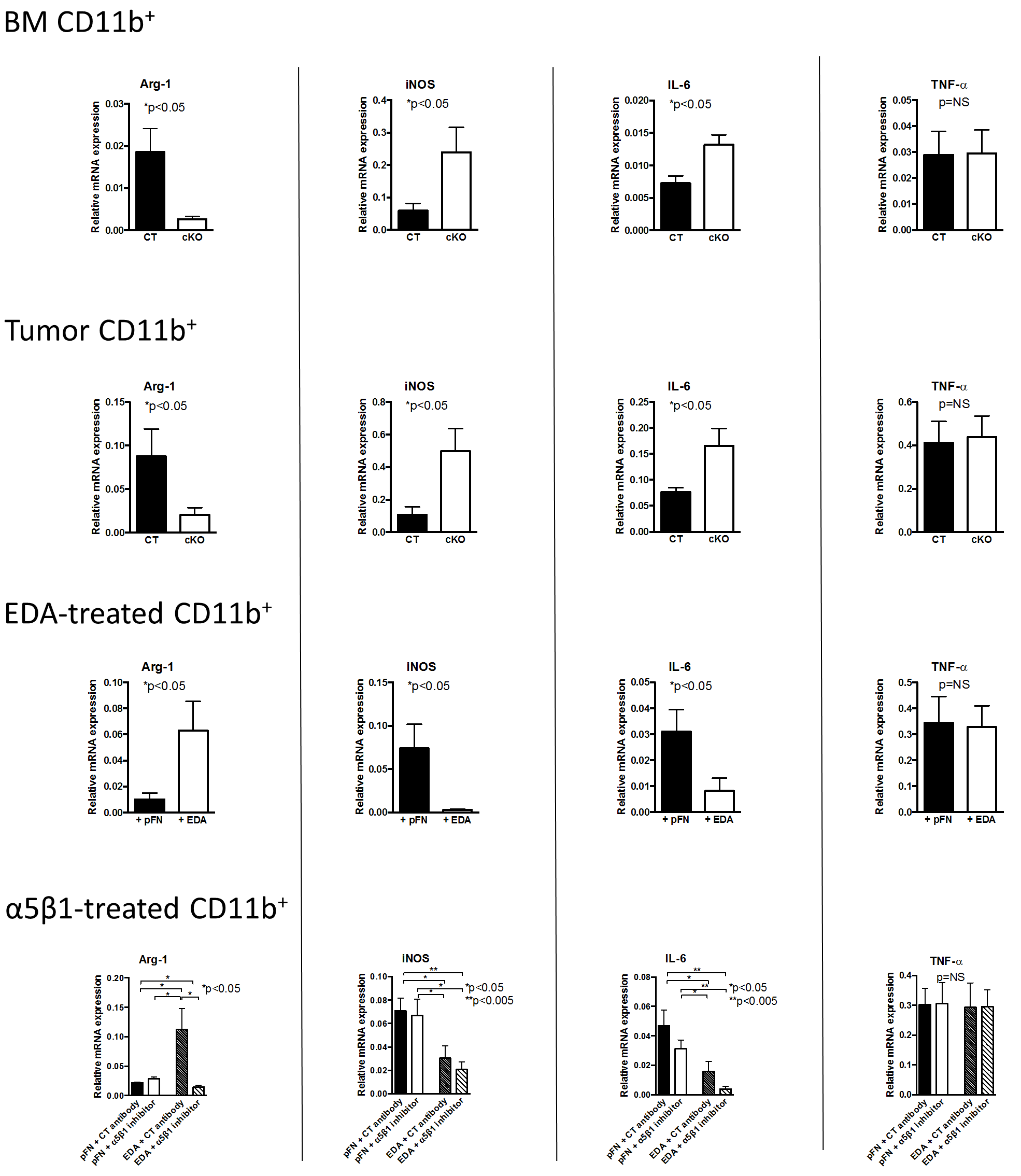

Supplement: S6 Fig — Cytokines were assessed using mRNA expression. Arginase-1 showed diminished mRNA expression in cKO bone marrow and tumors, but the expression could be stimulated by the addition of EDA but not pFN. Inhibition of integrin α5β1 reversed the effect of EDA (first column). iNOS and IL-6 both showed opposite expression patterns compared to arginase-1 in bone marrow, tumor, and EDA-treatment, but the EDA-effects could not be reversed by inhibiting integrin α5β1. Finally, TNFα was not influenced by the various conditions, n = 8/10, 5/7, 8/8, 8/8/8/8. Analysis was performed by t tests for the first three rows and ANOVA for the fourth row followed by t tests. Underlying data are provided in S1 Data. (TIF) [file pbio.1002562.s007.tif]

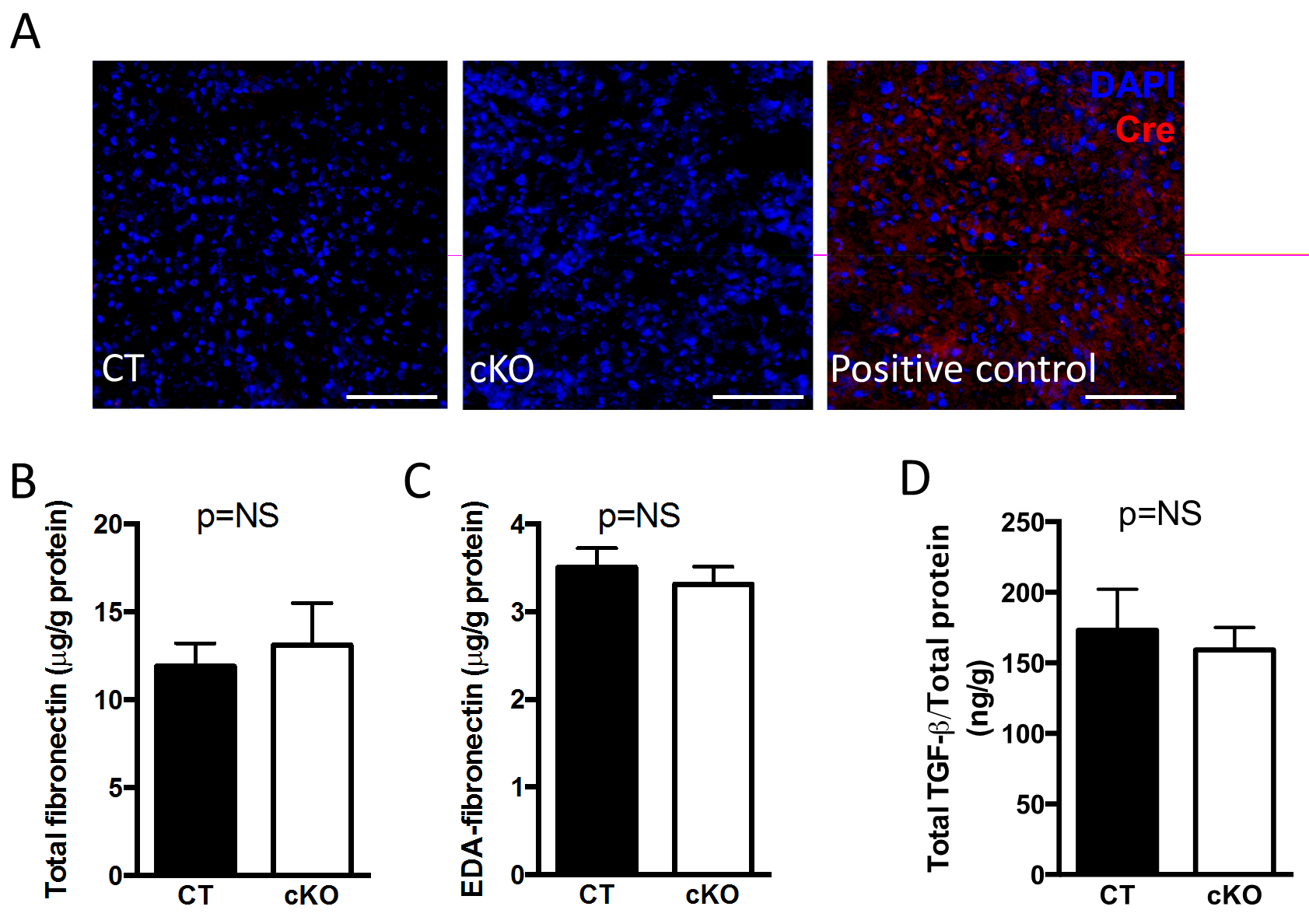

Supplement: S7 Fig — In order to determine whether the collagen α1(I) promoter has any activity in the liver, we stained liver sections from cKO animals against cre and compared to CT animals and to Mx-cre animals (marked as positive control). No staining was detected in collagen α1(I)-cre-harboring cKO animals (A); bars represent 100 μm. The total FN content of the liver was not affected (B), neither was the amount of EDA-containing FN (C), n = 9/12 for B and C. In line with these findings, total TGF-β was unchanged between CT and cKO animals (D), n = 7/6. Underlying data for B–D are provided in S1 Data. (TIF) [file pbio.1002562.s008.tif]
